# ﻿A further step towards the characterisation of *Terebellides* (Annelida, Trichobranchidae) diversity in the Northeast Atlantic, with the description of a new species

**DOI:** 10.3897/zookeys.1132.91244

**Published:** 2022-11-28

**Authors:** María Barroso, Juan Moreira, María Capa, Arne Nygren, Julio Parapar

**Affiliations:** 1 Departamento de Bioloxía, Universidade da Coruña, A Coruña, Spain Universidade da Coruña A Coruña Spain; 2 Departamento de Biología (Zoología) & Centro de Investigación en Biodiversidad y Cambio Global (CIBC-UAM), Facultad de Ciencias, Universidad Autónoma de Madrid, Madrid, Spain Universidad Autónoma de Madrid Madrid Spain; 3 Departament de Biologia, Universitat de les Illes Balears, Mallorca, Spain Universitat de les Illes Balears Mallorca Spain; 4 Sjöfartmuseet Akvariet, Göteborg, Sweden Sjöfartmuseet Akvariet Göteborg Sweden; 5 Institutionen för marina vetenskaper, Göteborgs Universitet, Göteborg, Sweden Göteborgs Universitet Göteborg Sweden

**Keywords:** DNA barcoding, DNA species delineation, identification key, integrative taxonomy, Northeast Atlantic, polychaetes, SEM, systematics

## Abstract

Several new species of genus *Terebellides* Sars, 1835 (Annelida, Trichobranchidae) have been recently described from the Northeast Atlantic Ocean after the detection of a large complex of species based on DNA sequence data from previous research. Some of those species (belonging to the so-called Group A) have already been described elsewhere. In this paper, we revise several *Terebellides* clades belonging to Groups B, C and D resulting in the identification of five nominal species: *Terebellidesgracilis* Malm, 1874, *Terebellidesatlantis* Williams, 1984, *Terebellideswilliamsae* Jirkov, 1989, *Terebellidesirinae* Gagaev, 2009, and *Terebellidesshetlandica* Parapar, Moreira & O’Reilly, 2016, plus one new species described here as *Terebellideslavesquei***sp. nov.** All these species are characterised by a combination of morphological features complemented with a nucleotide diagnostic approach (specific COI nucleotides in the alignment position). Morphological characters used to discriminate between taxa refer to the branchial shape, presence/absence of ciliated papillae dorsal to thoracic notopodia and the morphology of thoracic and abdominal uncinal teeth. An updated identification key to all described species of this genus in NE Atlantic waters is also included.

## ﻿Introduction

The genus *Terebellides* Sars, 1835 (Annelida) is distinguishable from other members of Trichobranchidae by the unique mid-dorsal stalk bearing the characteristic branchial lobes, provided with packed branchial lamellae. This taxon is morphologically homogenous and boundaries between species are difficult to assess because relevant characters rely on some microscopic details. These include features of branchiae, chaetae and uncini that need examination with Scanning Electron Microscopy (Parapar et al. 2016c, 2020a; Lavesque et al. 2019). In addition, further work is still needed to assess morphological intra- and interspecific variability for some characters.

A recent comprehensive molecular survey and a set of analytical methods (Nygren et al. 2018) revealed that the actual diversity of *Terebellides* is much higher than expected increasing from 5 to 25 the total number of species present in the NE Atlantic. Furthermore, molecular-based phylogenetic analyses by Nygren et al. (2018) facilitated the search of diagnostic characters. Thus, three nominal species have been identified, including the type species, *Terebellidesstroemii* Sars, 1835, and six have been described new to science (Parapar et al. 2020a). All these belonged to the so-called Group A (sensu Nygren et al. 2018). In addition, Lavesque et al. (2019) described eight new species from France based in a molecular survey as well. Many of these recently described species had been usually misidentified as *T.stroemii*; this taxon was previously thought as having a cosmopolitan distribution and resulted therefore in many species being overlooked worldwide.

Nygren et al. (2018) stablished four main groups of species: A, B, C, and D, which differ from each other by several morphological characters. Parapar et al. (2020b) studied group A species that are characterised by greater overall body length (10.0–50.0 mm), branchial lobes almost completely fused with ventral lobes that were partially or totally obscured, and the presence of papillae on margins of branchial lamellae in some species.

The aim of the present study is the morphological and molecular characterisation of members of Groups B, C, and D after Nygren et al. (2018). A total of five nominal species was identified and a new species is here described as *Terebellideslavesquei* sp. nov.

## ﻿Materials and methods

This paper is based on the study of 215 *Terebellides* specimens belonging to Groups B, C, and D as defined in Nygren et al. (2018) (Table 1); five correspond to previously described species and one is new to science. This material is deposited in the Zoological Museum Bergen (**ZMBN**, Bergen, Norway), Goteborg Natural History Museum (**GNM**, Goteborg, Sweden), and the Norwegian University of Science and Technology, Science Museum (**NTNU-VM**, Trondheim, Norway; Bakken et al. 2020).

**Table 1.** Comparison of discriminating taxonomic characters of the species studied in this work. Cells in italics show discriminatory characters of each subgroup. ^(1)^ sensu Parapar et al. (2016a); ^(2)^ sensu Parapar et al. (2020b); ^(3)^ sensu Parapar et al. (2020a); ^(4)^ dominant trend in bold; ^(5)^ Skagerrak.

Groups		B			C	D	
Putative species sensu Nygren et al. (2018)		1	5	16	24	2	3
Species (as reported/described herein)		*T.shetlandica* Parapar, Moreira & O’Reilly, 2016	*T.lavesquei* sp. nov.	*T.atlantis* Williams, 1984	*T.irinae* Gagaev, 2009	*T.williamsae* Jirkov, 1989	*T.gracilis* Malm, 1874
Branchiae	type ^(1)^	3	2	3	4	2	2
	papillae on lamellae edge	no	no	no	no	no	no
Thorax	ciliated papilla dorsal to notopodium	no (?)	no (?)	no (?)	no (?)	*yes*	*yes*
	chaetiger with geniculate chaetae	TC 6	TC 6	TC 6	TC 6	TC 6	TC 6
	uncini type ^(2)^	*4*	*3*	*3*	*3*	*1*	*1*
Abdomen	uncini type ^(3)^	*2*	*2*	*2*	*2*	*1A*	*1A*
Bathymetry – Above (A) / Below (B) 200 m depth ^(4)^		**A**/B	A/B	B	B	B	B
Distribution – North (N) /South (S) of 60°N ^(4)^		N/**S**^(5)^	**N**/S	N	N	**N**/S^(5)^	N/**S**^(5)^

These specimens are part of a large collection of *Terebellides* specimens (table S1 in Nygren et al. 2018) mostly collected in the Norwegian and Swedish continental shelf but also from the Irish and Celtic seas, North Sea, Barents Sea, Greenland Sea, South Icelandic coast, and the Arctic Ocean.

Light microscope photographs were done using an Olympus SZX12 stereomicroscope equipped with an Olympus C-5050 digital camera. Line drawings were made with a Wacom CTL-4100K-S pen tablet based on photographs and observations made with an Olympus BX40 stereomicroscope. Specimens for Scanning Electron Microscopy (SEM) were prepared by critical point drying, covered with gold and examined and photographed under a JEOL JSM-6400 electron microscope at the Servizos de Apoio á Investigación (**SAI**, Universidade da Coruña, Spain).

For staining procedures, 10 mg of Methyl Green (MG) colourant were dissolved in 5 ml of 20% ethanol and specimens were held in there for 30 s. MG staining patterns and thoracic uncini morphology were characterised based on the classifications proposed by Schüller and Hutchings (2010, 2013) and Parapar et al. (2020b), respectively; only specimens of similar/comparable size were considered.

For each species, the list of the museum registration numbers and collection details (geographic area, locality, coordinates, depth, collecting date and habitat) is provided in Suppl. material 1. Unless specified, each registration number holds a single specimen; associated GenBank DNA sequence accession numbers are provided in Suppl. material 2.

The correspondence between species numerals (Nygren et al. 2018) and names is as follows: species 1 –*Terebellidesshetlandica* Parapar, Moreira & O’Reilly, 2016; species 5 – *Terebellideslavesquei* sp. nov.; species 16 – *Terebellidesatlantis* Williams, 1984; species 24 – *Terebellidesirinae* Gagaev, 2009; species 2 – *Terebellideswilliamsae* Jirkov, 1989; species 3 – *Terebellidesgracilis* Malm, 1874.

The present study deals with the main *Terebellides* groups B, C, and D, proposed by Nygren et al. (2018) after phylogenetic analyses of nuclear (28S rDNA and internal transcriber spacer 2, ITS2) and mitochondrial (cytochrome C oxidase I and 16S r DNA) markers from specimens of Northeast Atlantic (**NEA**) *Terebellides*, representing a follow-up to Parapar et al. (2020a) who characterised the species within Group A. In this way, additional analyses with only the COI dataset have been performed in order to assess diagnostic nucleotides for each of the species and the genetic distances between them. Phylogenetic analyses of COI *Terebellides* sequences in GenBank generated by Nygren et al. (2018) and Lavesque et al. (2019) were performed, using *Trichobranchusroseus* (Malm, 1874), *Polycirrus* sp., and Pistacf.cristata (Müller, 1776) as outgroups (Nygren et al. 2018). Methodology followed that described by Parapar et al. (2020a) and included alignment of 471 sequences with MAFFT version 7.017 (Katoh et al. 2002), calculation of the best substitution model (TVM+F+I+G4), according to Bayesian information criterion – BIC with IQTREE version 1.6.11 (Nguyen et al. 2015). Maximum likelihood phylogenetic analyses were run in IQTREE version 1.6.11 (Nguyen et al. 2015), with ultrafast bootstrap (Hoang et al. 2018). Unequivocal nucleotide diagnostic characters are provided as the positions in the alignment, shown in Suppl. material 2.

The most distinctive taxonomic morphological characters for *Terebellides* include morphology of branchiae (sensu Parapar et al. 2016a), type of thoracic uncini (sensu Parapar et al. 2020b) and abdominal uncini (sensu Parapar et al. 2020a); Methyl Green (MG) staining pattern (sensu Schüller and Hutchings 2010, 2013) and geographic and bathymetric distribution data are also useful to discern species. Regarding branchiae, Parapar et al. (2016a) proposed four types: type 1, with large lobes almost completely fused; type 2, with lobes fused ~ 50% of their length; type 3, with lobes only fused at base; and type 4, with small lobes not fused and reduced dorsal lobes. Parapar et al. (2020a, b) also defined four types of thoracic uncini and three types of abdominal uncini based on the rostrum vs. capitium length ratio (RvC), and the relative size of the capitium teeth. In the species studied here, three types of thoracic uncini have been identified: type 1 – RvC = 2(3)/1, capitium with two or three large teeth and subsequent ones much smaller; type 3 – RvC = 1/1, capitium with four or five mid-sized teeth followed by slightly smaller teeth; type 4 – RvC = 1/ 1, capitium with 5–7 small teeth and remaining ones similar in size at least in two rows. Two types of abdominal uncini were also identified: type 1A – RvC = 1/0.7, capitium with 3–5 large teeth in first row and one or two in a second row; type 2 – RvC = 1/0.9, capitium with four or five teeth and remaining ones smaller.

Schüller and Hutchings (2010, 2013) defined several types of Methyl Green staining patterns. The patterns observed in the species studied here are similar to the following patterns: pattern 1, segments (SG) 1–6 solid and SG 7–14 striped; pattern 2, SG 1–5 solid, SG 6 white and SG 7–14 striped; and pattern 9, SG 1–5 solid, SG 3 with J-shape glandular region, SG 6 dark solid and SG 7–18 striped.

### ﻿Abbreviations used in text, table, and figures:

**abl** anterior branchial lobe (lobe #5);

**bdl** branchial dorsal lobes;

**bdltp** branchial dorsal lobe terminal papilla;

**bf** branchial filament;

**bvl** branchial ventral lobes;

**cap** capitium;

**cop** copepod;

**cr** ciliary row;

**ct** ciliary tuft;

**dpn** dorsal projection of notopodium;

**gc** geniculate chaetae;

**MG** Methyl Green;

**NEA** Northeast Atlantic;

**nc** notochaetae;

**ooc** oocytes;

**RvC** rostrum vs. capitium length ratio;

**SEM** Scanning Electron Microscope;

**SG** segment;

**STM** stereomicroscope;

**TC** thoracic chaetiger;

**tdp** thoracic dorsal papilla;

**tll** thoracic lateral lappets;

**tm** tentacular membrane;

**TU** thoracic unciniger;

**wTC** white thoracic chaetiger.

## ﻿Systematic account

**Figure 1.**
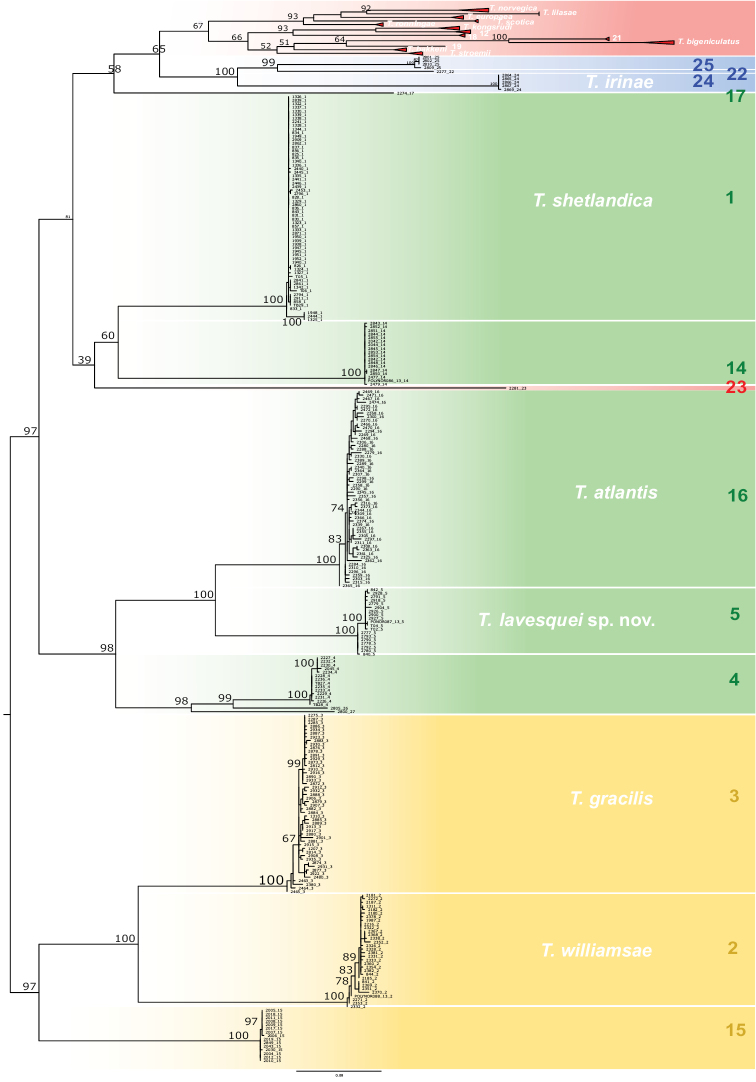
Phylogenetic tree after Maximum Likelihood analyses on a dataset of all COI sequences in Nygren et al. (2018) and Lavesque et al. (2019). Bootstrap support values above nodes. Species other than members of Groups B, C, and D are collapsed.

Five lineages of the *Terebellides* Groups B, C, and D (sensu Nygren et al. 2018) were identified as nominal species already reported in the Northeast Atlantic: *Terebellidesgracilis* Malm, 1874, *T.atlantis* Williams, 1984, *T.williamsae* Jirkov, 1989, *T.irinae* Gagaev, 2009, and *T.shetlandica* Parapar, Moreira & O’Reilly, 2016. In addition, one of the lineages did not match any of the previously known *Terebellides* species and is herein describes as new: *Terebellideslavesquei* sp. nov. (Fig. 1).

Following Nygren et al. (2018) nomenclature and grouping, species have been here grouped as follows: Group B – species 1, 5, and 16, Group C – species 24, and Group D – species 2 and 3; description of each taxon will follow this order. Species 17, 27 (Group B), and 22 (Group C) are represented by 1–4 specimens each (see appendix S36 in Nygren et al. 2018) and are pending formal description until more material is available. Species 4, 14, and 26 (Group B), 25 (Group C), and 15 (Group D) will be described by D. Gaeva and I. Jirkov (Shirshov Institute of Oceanology, Russia). For correspondence between species names and numerals see below.

### Family Trichobranchidae Malmgren, 1866

#### ﻿Genus Terebellides

Taxon classificationAnimaliaTerebellidaTrichobranchidae

Sars, 1835 emended by Schüller & Hutchings, 2013

0EC31A17-926F-5013-A4A6-C8E2E527CA25

##### Type species.

*Terebellidesstroemii* Sars, 1835, redescribed by Parapar and Hutchings (2014) and neotype deposited.

#### ﻿ Terebellides

Taxon classificationAnimaliaTerebellidaTrichobranchidae

Group B (sensu Nygren et al. 2018)

612825B9-1C42-512D-AD48-8660C27B8944

Figs 2 , 3 , 4 , 5 , 6 , 7 , 8 , 9 , 10 , 11 , 12

##### Description.

The morphological features shared by all examined species in Group B in this paper (clades 1, 5, and 16) are itemised below. Some of these are also shared by Groups A, C, and D as defined in Nygren et al. (2018) (see Remarks below). Clades 4, 14, and 26 will be studied elsewhere; formal descriptions of clades 17 and 27 will wait until more material is available.

**Figure 2.**
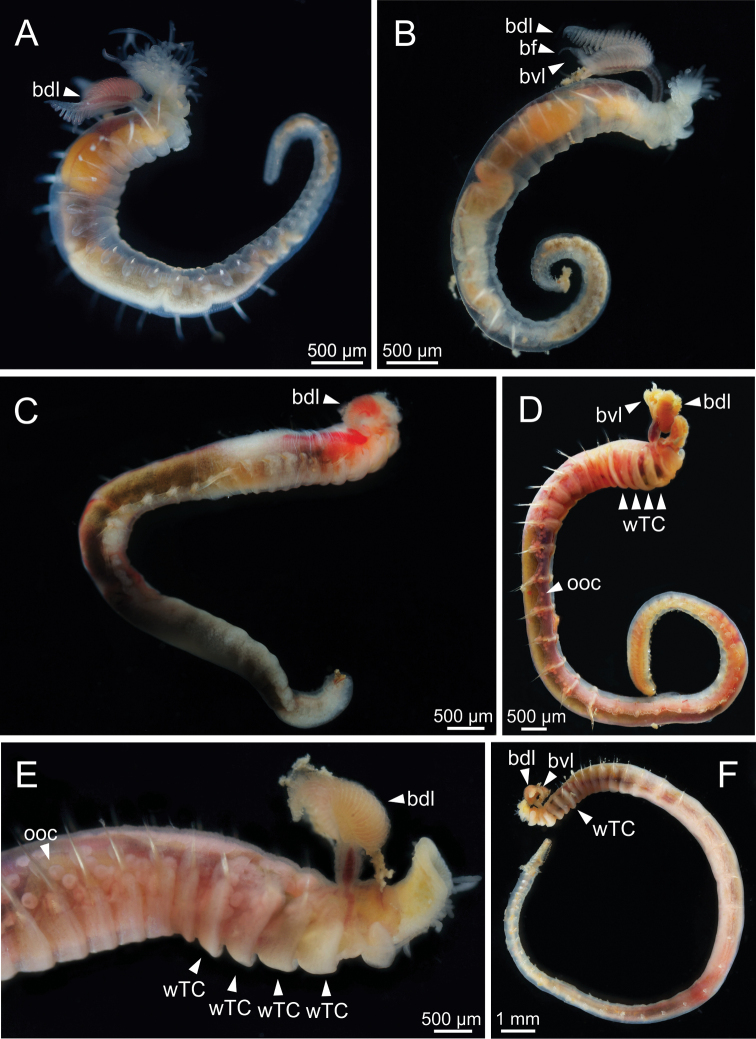
STM photographs of live specimens of several *Terebellides* species (non-type specimens) **A, B***Terebellidesshetlandica* Parapar, Moreira & O’Reilly, 2016 (species 1; **A** ZMBN116171 **B** ZMBN116181) **C***Terebellideslavesquei* sp. nov. (species 5; GNM15112) **D, E***Terebellideswilliamsae* Jirkov, 1989 (species 2; **D** GNM15108 **E** GNM15109) **F***Terebellidesgracilis* Malm, 1874 (species 3; GNM15111). Abbreviations: bdl – branchial dorsal lobe; bf – branchial filament; bvl – branchial ventral lobe; ooc – oocytes; wTC – white thoracic chaetiger.

**Figure 3.**
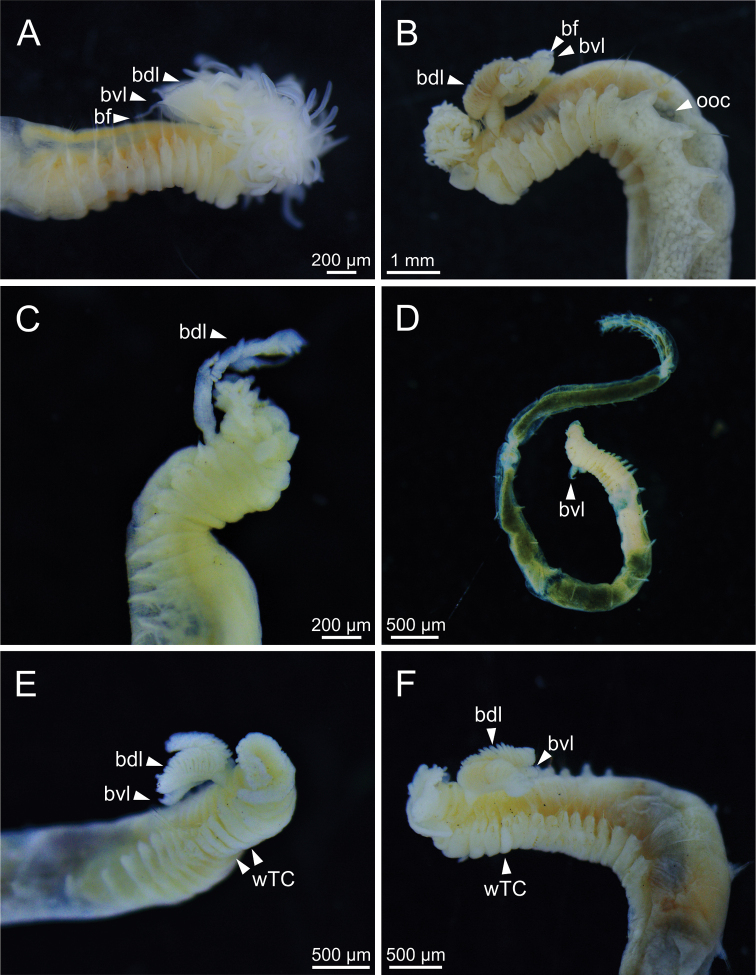
STM photographs of several *Terebellides* species (**A, C–F** non-type specimens) **A***Terebellidesshetlandica* Parapar, Moreira & O’Reilly, 2016 (species 1; ZMBN116186) **B***Terebellideslavesquei* sp. nov. (species 5; holotype, ZMBN116322) **C***Terebellidesatlantis* Williams, 1984 (species 16; ZMBN116472) **D***Terebellidesirinae* Gagaev, 2009 (species 24; ZMBN116498) **E***Terebellideswilliamsae* Jirkov, 1989 (species 2; ZMBN116269) **F***Terebellidesgracilis* Malm, 1874 (species 3; ZMBN116283). Abbreviations: bdl – branchial dorsal lobe; bf – branchial filament; bvl – branchial ventral lobe; ooc – oocytes; wTC – white thoracic chaetiger.

***Body appearance*.** Complete individuals ranging from 5.0–35.0 mm in length. Body tapering posteriorly with segments increasingly shorter and crowded towards pygidium. Prostomium compact; large tentacular membrane surrounding mouth (Figs 2B, 3B, C), with typical buccal tentacles with expanded tips (Figs 2A, B, 3A, B). SG 1 as an expanded structure below tentacular membrane in a lower lip.

**Figure 4.**
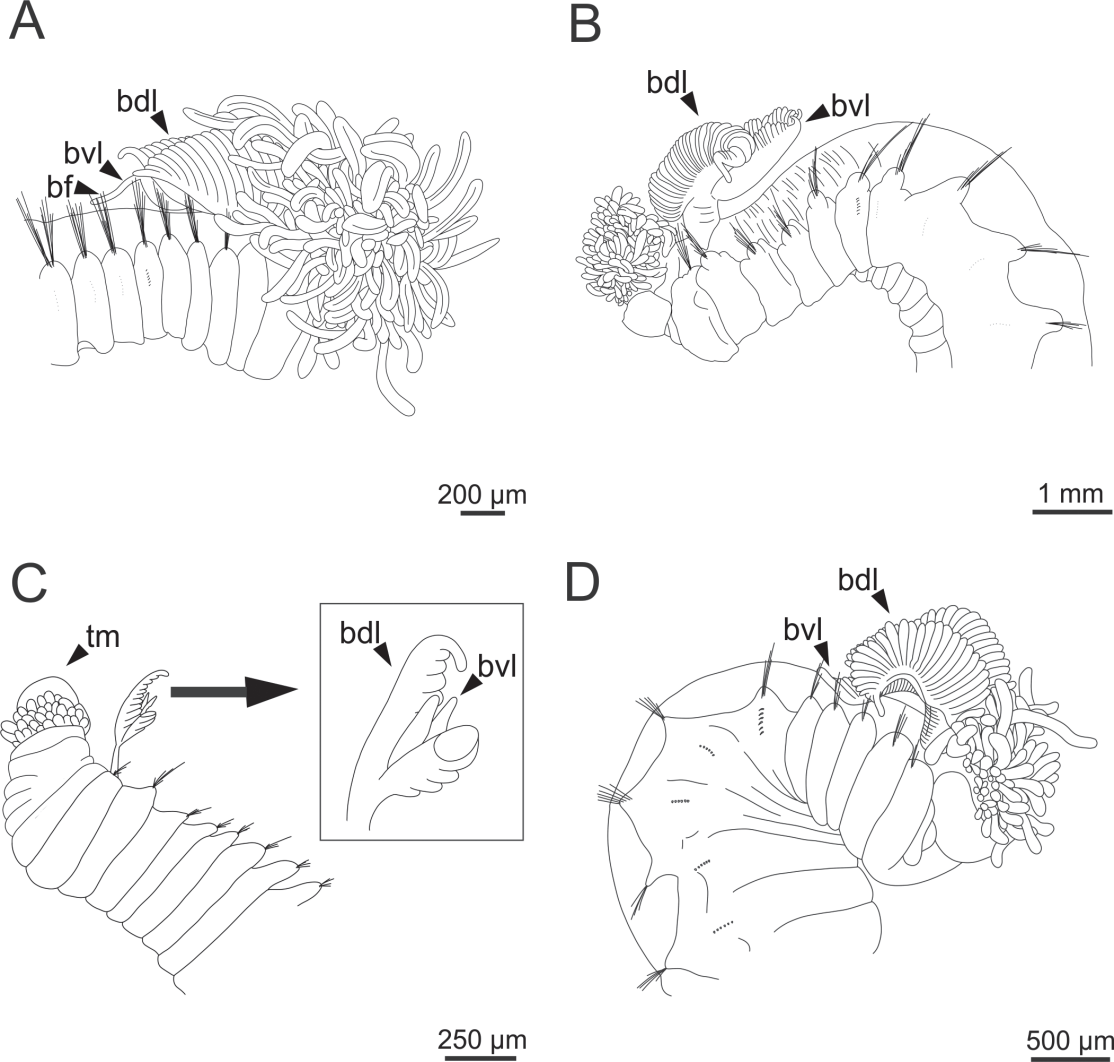
Line drawings of several *Terebellides* species (**A, C, D** non-type specimens) **A***Terebellidesshetlandica* Parapar, Moreira & O’Reilly, 2016 (species 1; ZMBN116186), anterior end, right lateral view **B***Terebellideslavesquei* sp. nov. (holotype; ZMBN116322), anterior end, left lateral view **C***Terebellidesirinae* Gagaev, 2009 (species 24; ZMBN116498), anterior end, ventral view **D***Terebellidesgracilis* Malm, 1874 (species 3; ZMBN116283), anterior end, right lateral view. Abbreviations: bdl – branchial dorsal lobe; bf – branchial filament; bvl – branchial ventral lobe; tm – tentacular membrane.

***Branchiae*.** Branchiae arising as single structure from SG 3, with a single stalked mid-dorsal stem (Figs 2A, B, 3B, C, 4B), lobes not fused or partially fused, ventral ones obscured or not by dorsal ones (Figs 2A, B, 3A–C, 4A, B). Dorsal lobes ending posteriorly in short terminal papilla and ventral lobes ending in long filaments (Figs 2B, 3A, B). Anterior projection of dorsal lobes (fifth lobe) normally absent but present only in clade 5 (Fig. 3B). Posterior end of dorsal lobes reaching TC 4 (Figs 2A, B, 3A, B). Branchial lamellae provided with several parallel rows of cilia in inner face (Fig. 6C) and ciliary tufts not observed. Ciliary papillae absent in branchial lamellae margin.

**Figure 5.**
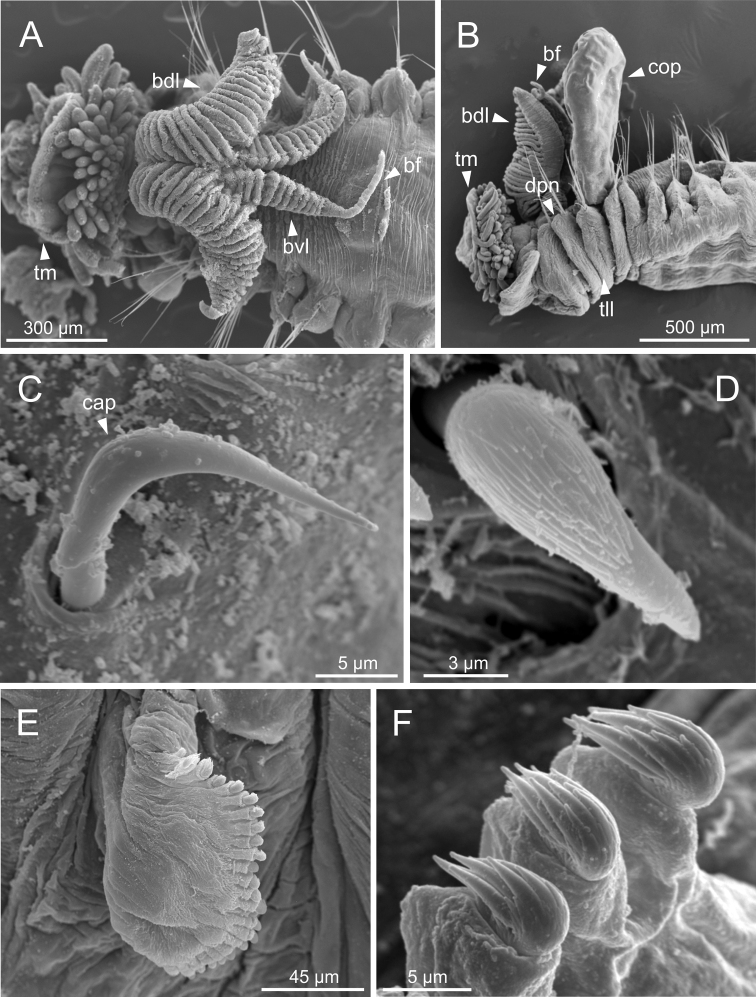
*Terebellidesshetlandica* Parapar, Moreira & O’Reilly, 2016 (species 1; non-type specimens, ZMBN116181, ZMBN116204 and ZMBN116219), SEM micrographs **A** anterior end, dorsal view **B** anterior end, left lateral view **C**TC 6 (TU1), geniculate chaeta **D** thoracic uncinus **E** abdominal neuropodium **F** abdominal uncini. Abbreviations: bdl – branchial dorsal lobe; bf – branchial filament; bvl – branchial ventral lobe; cap – capitium; cop – copepod; dpn – dorsal projection of notopodium; TC – thoracic chaetiger; tll – thoracic lateral lobes; tm – tentacular membrane; TU – thoracic unciniger.

***Thorax*.** Eighteen pairs of notopodia (SG 3–20), those of TC 1 ca. as long as subsequent ones or slightly shorter (Figs 2A, 3A–C). Lateral lappets and dorsal projections of notopodia in anterior thoracic chaetigers with different degree of development depending on size and preservation conditions, but both more conspicuous on TC 1–4 (Fig. 3A–C). All notochaetae as simple capillaries (Fig. 5B). Size of notochaetae of TC 1 similar to subsequent ones. Neuropodia as sessile pinnules from TC 6 to body end, with uncini in single or double rows, from TC 7 throughout. Neuropodia on TC 6, provided with several sharply bent, acute-tipped, geniculate chaetae with minute teeth forming a capitium only visible with SEM (Figs 5C, 7A, 8C). From TC 7, neuropodia with one or several rows of uncini per torus (Figs 5D, 7B, C, 8D), with long shafted denticulate hooks, with large main fang (rostrum) longer than upper crest of teeth (capitium), rostrum/capitium length ratio of ~ 2:1, capitium composed by several teeth above main fang of decreasing length (Figs 5D, 7B, 8D).

**Figure 6.**
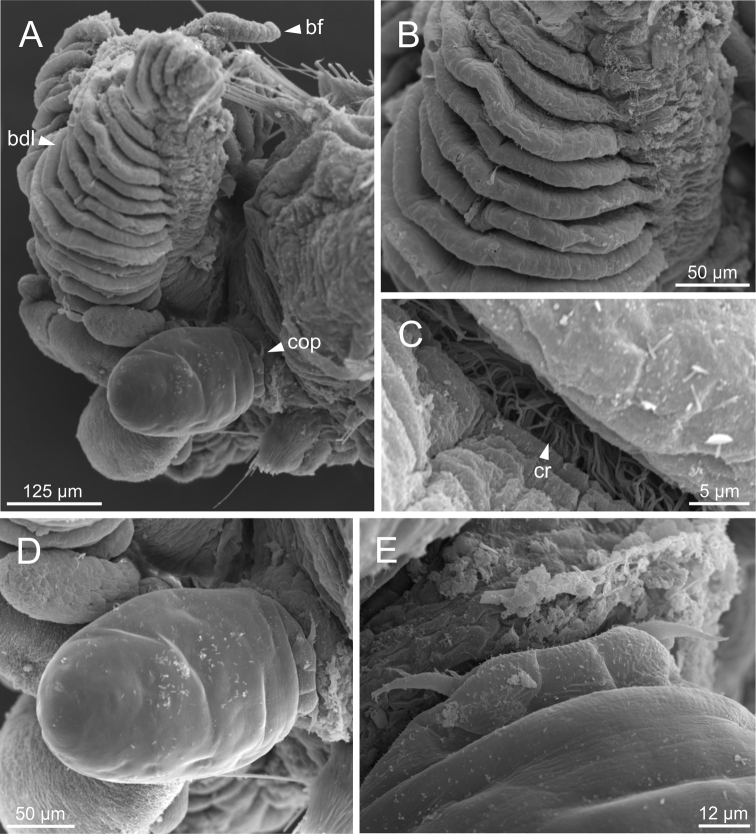
*Terebellideslavesquei* sp. nov. (non-type specimen, ZMBN116332), SEM micrographs **A** anterior end, left lateral view **B** branchial lamellae, detail **C** ciliary row, detail **D** copepod **E** copepod, anterior end. Abbreviations: bdl – branchial dorsal lobe; bf – branchial filament; cop – copepod; cr – ciliary row.

**Figure 7.**
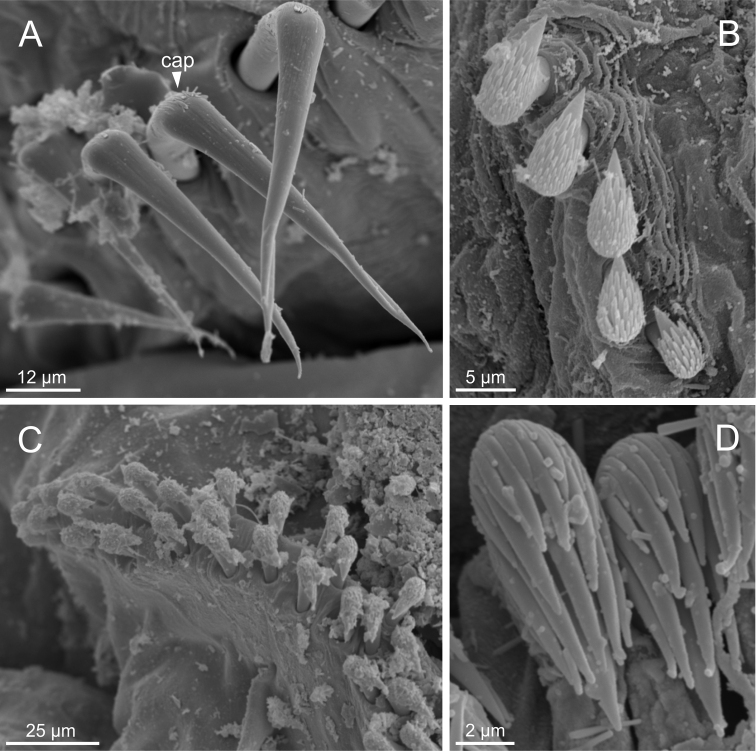
*Terebellideslavesquei* sp. nov. (non-type specimens, NTNU–VM61387 and ZMBN116332), SEM micrographs **A**TC 6 (TU1), geniculate chaetae **B** thoracic uncini **C** double row of thoracic uncini **D** abdominal uncini. Abbreviations: cap – capitium; TC – thoracic chaetiger; TU – thoracic unciniger.

**Figure 8.**
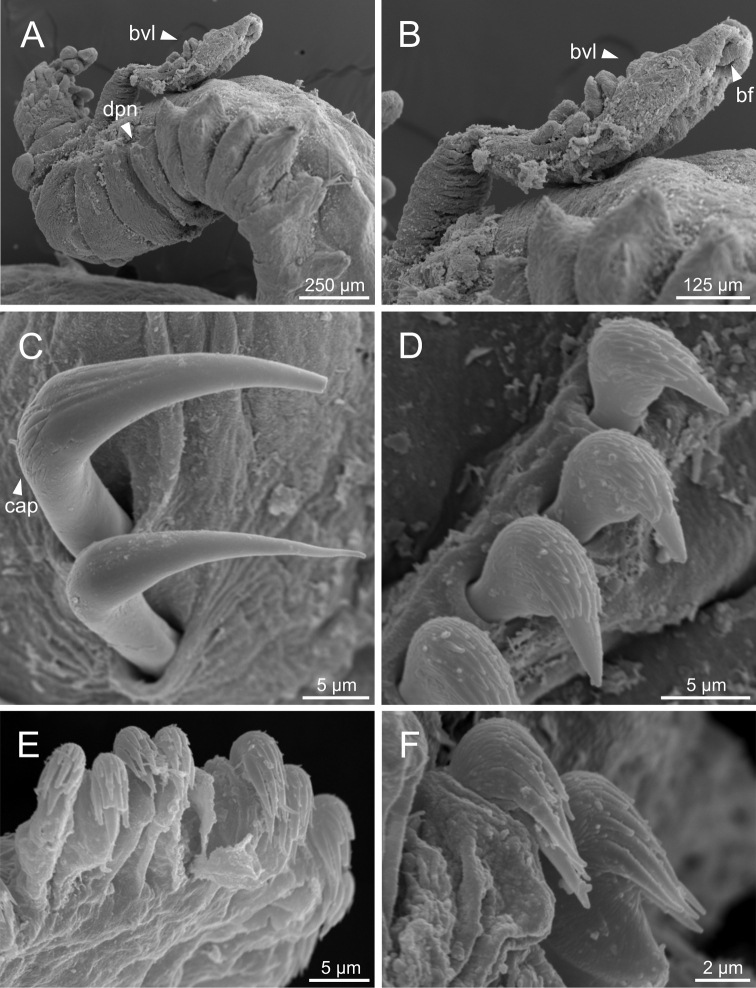
*Terebellidesatlantis* Williams, 1984 (species 16; non-type specimens, ZMBN116454 and ZMBN116459), SEM micrographs **A** anterior end, left lateral view **B** branchiae, detail **C**TC 6 (TU1), geniculate chaetae **D** thoracic uncini **E** abdominal neuropodium **F** abdominal uncini. Abbreviations: bf – branchial filament; bvl – branchial ventral lobe; cap – capitium; dpn – dorsal projection of notopodium; TC – thoracic chaetiger; TU – thoracic unciniger.

***Abdomen and pygidium*.** Approximately half as long as thorax and progressively thinner (Fig. 2A–C). Neuropodia ranging from 18–34 chaetigers and forming erect pinnules (Figs 5E, 8E) with several uncini per torus, number depending on specimen size. Uncini provided with several teeth above rostrum surmounted by a capitium composed of several teeth of decreasing length (Figs 5F, 7D, 8F). Pygidium blunt, as funnel-like depression.

***Colour pattern*.** Colour in preserved specimens whitish or pale brown (Fig. 3A–C). MG staining pattern characterised by 1) compact green colourant in SG 1–6, then turning into striped pattern in SG 7–14 and fading in following segments or 2) by compact green colourant in SG 1–6, J-shaped glandular region in SG 3–5, and striped pattern in SG 7–14 (Fig. 12).

##### Remarks.

Among the aforementioned characters, branchial features might serve to distinguish most of Group B species from those of Groups A, C, and D. Those include size of branchial lobes, lobes not fused, presence of long filaments on ventral ones, and presence of ciliary rows on branchial lamellae. Other taxa described or reported worldwide bear the same type of branchiae (type 3), including *Terebellidesehlersi* McIntosh, 1885, *T.intoshi* Caullery, 1915, *T.lobatus* Hartman & Fauchald, 1971, *T.mundora* Hutchings & Peart, 2000 and *T.sepultura* Garraffoni & Lana, 2003 (Parapar et al. 2016a, b).

#### ﻿ Terebellides shetlandica

Taxon classificationAnimaliaTerebellidaTrichobranchidae

Parapar, Moreira & O’Reilly, 2016

37ADFC8C-713C-5C8A-8645-BBC870D0A3D4

Figs 2A, B , 3A , 4A , 5 , 9 , 10A , 11 , 12 , Table 1 , Suppl. materials 1 , 2

Terebellides shetlandica Parapar, Moreira & O’Reilly, 2016a: 211–225, figs 1–9, 11.
Species 1 – Nygren et al. 2018: 18–22, figs 6, 10.

##### Material examined.

30 specimens (Suppl. material 1), Skagerrak (GNM14640); Swedish coast (ZMBN116171, ZMBN116181, ZMBN116185, ZMBN116186, ZMBN116187, ZMBN116188, ZMBN116191, ZMBN116192, ZMBN116193, ZMBN116196, ZMBN116198, ZMBN116200, ZMBN116201, ZMBN116202, ZMBN116203, ZMBN116204, ZMBN116206); Norwegian coast (ZMBN116207, ZMBN116208, ZMBN116214, ZMBN116216, ZMBN116219, ZMBN116220, ZMBN116221, ZMBN116226, ZMBN116227, ZMBN116228, ZMBN116235, ZMBN116242).

##### GenBank accession numbers of material examined (COI).

MG024894, MG024895, MG024896, MG024897, MG024898, MG024899, MG024900, MG024901, MG024902, MG024903, MG024904, MG024905, MG024906, MG024907, MG024908, MG024909, MG024910, MG024911, MG024912, MG024913, MG024914, MG024915, MG024916, MG024917, MG024918, MG024919, MG024920, MG024921, MG024922, MG024923, MG024924, MG024925, MG024926, MG024927, MG024928, MG024929, MG024930, MG024931, MG024932, MG024933, MG024934, MG024935, MG024936, MG024937, MG024938, MG024939, MG024940, MG024941, MG024942, MG024943, MG024944, MG024945, MG024946, MG024947, MG024948, MG024949, MG024950, MG024951, MG024952, MG024953, MG024954, MG024955, MG024956.

##### Diagnostic features of studied material.

**Figure 9.**
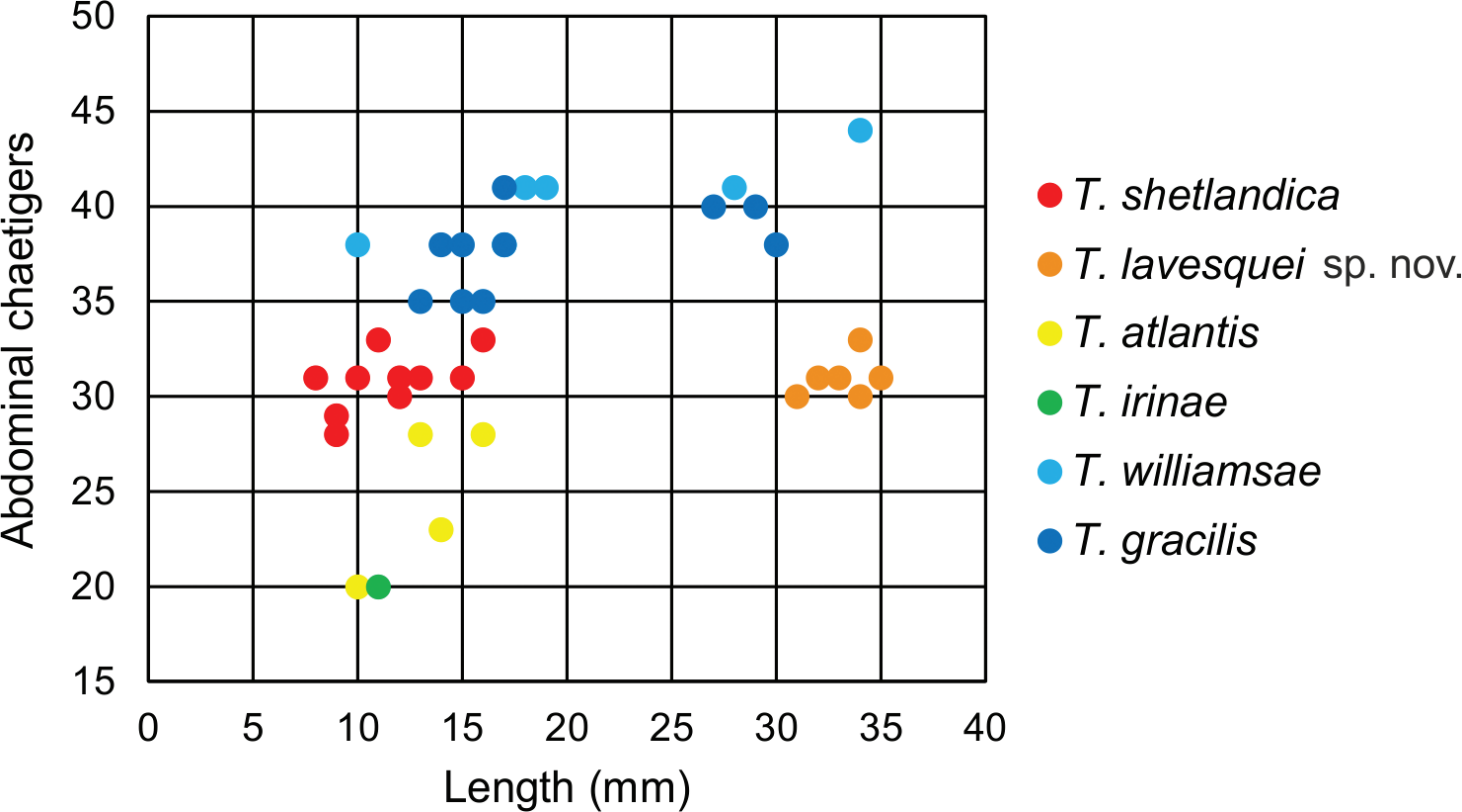
Relationship between number of abdominal chaetigers and body length (complete specimens considered except for *T.irinae*).

Complete individuals ranging from 5.0–16.0 mm in length (Fig. 9). Branchial dorsal lobes lamellae provided with well-developed papillary projections and branchial ventral lobes provided with long filaments, ranging from 175.0–225.0 µm in length (Figs 2A, B, 4A, 5A, B). Between 22–26 lamellae on dorsal lobes (Fig. 5A, B). Lateral lappets present on TC 1–4; dorsal projections of thoracic notopodia on TC 2 and TC 3 (Fig. 5B). Geniculate chaetae in TC 5, acutely bent, with poorly marked capitium (Fig. 5C). Ciliated papilla dorsal to thoracic notopodia not observed. From TC 7, neuropodia with one row of type 4 thoracic uncini per torus, with rostrum/capitium length ratio of ~ 2:1 and capitium with a first row of small teeth, followed by several smaller teeth (Fig. 5D). Abdomen with 25–34 pairs of neuropodia with type 2 uncini (Fig. 5E, F). Copepods attached to body surface in three specimens (Fig. 5B).

##### Colour pattern.

MG staining pattern characterised by compact green colourant in SG 1–6, then turning into striped pattern in SG 7–14 and fading in following segments (Fig. 12). Similar to pattern 1.

##### Nucleotide diagnostic features.

All sequences of *Terebellidesshetlandica* share and are distinguished from other available *Terebellides* sequences in unique combinations of nucleotides (underlined) at the given position of our alignment: 78–98: CCAACCCGGAGCCTATTTAGGT, 186–192: CGGAAAC, 210–219: GCTAGGCGCC, 228–234: GGCATTC, 264–276: TCTCCCGCCTGCC, 288– 292: CGTT, 306: C, 333–342: CGTCTACCCT, 351–369: AGACAATATGGCACACGCC, 381–402: AGATCTGGCTATTTTCTCCCTA, 453–459: AGTAATA, 511–522: TCAGCTATAATC, 535–558: TTACTTCTTTCTCTGCCAGTTCTG.

##### Type locality.

NW Hutton Oilfield, between Shetland Islands and Norway, 61°10'N, 01°12'E (Parapar et al. 2016a).

##### Distribution and bathymetry.

**Figure 10.**
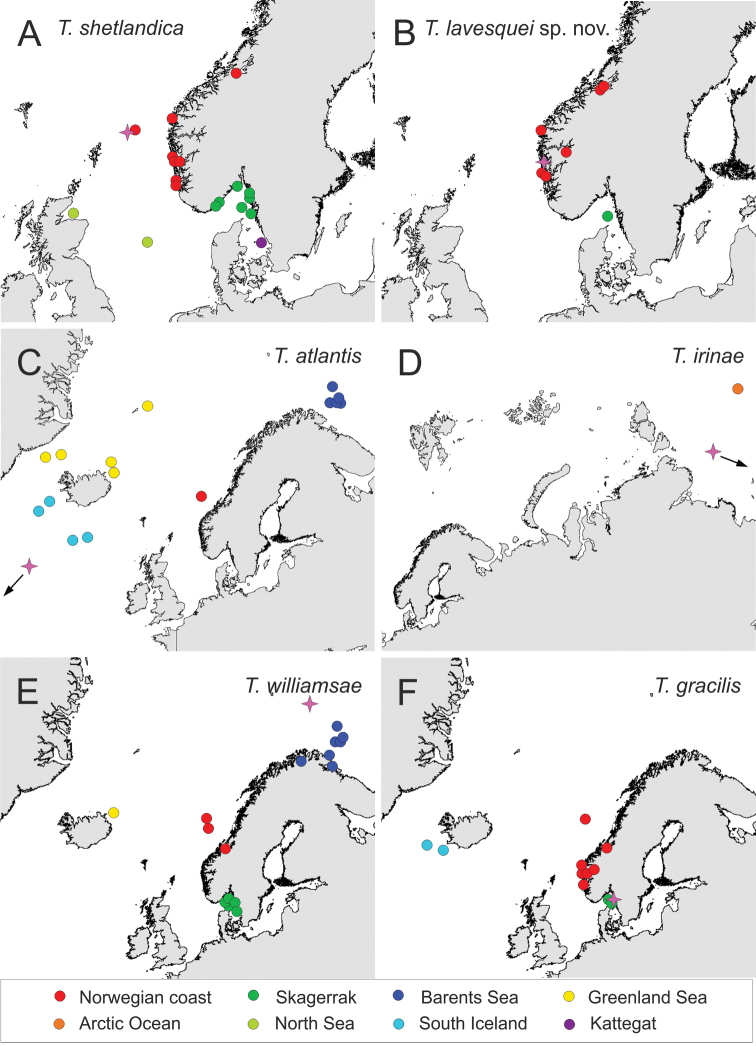
Geographic distribution of species of *Terebellides* in Northeast Atlantic Ocean **A***Terebellidesshetlandica* Parapar, Moreira & O’Reilly, 2016 **B***Terebellideslavesquei* sp. nov. **C***Terebellidesatlantis* Williams, 1984 **D***Terebellidesirinae* Gagaev, 2009 **E***Terebellideswilliamsae* Jirkov, 1989 **F***Terebellidesgracilis* Malm, 1874. Pink star denotes the type locality of each taxon.

**Figure 11.**
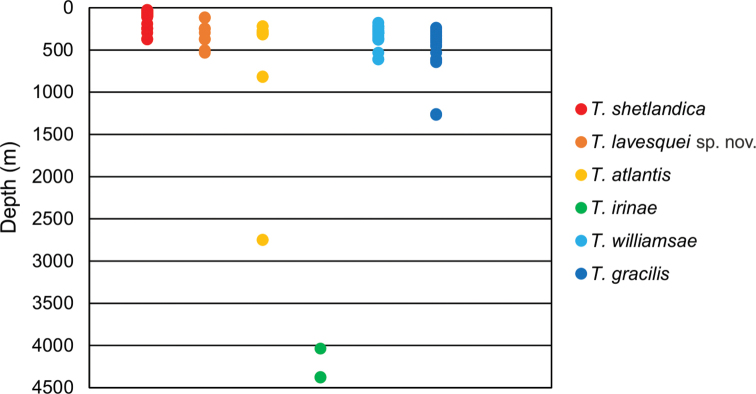
Bathymetric distribution of *Terebellides* species studied in this work.

Norwegian coast and shelf, North Sea, Skagerrak, Kattegat; 25–375 m deep; 92.7% of specimens present at depths below 200 m (Figs 10A, 11, Suppl. material 1).

**Figure 12.**
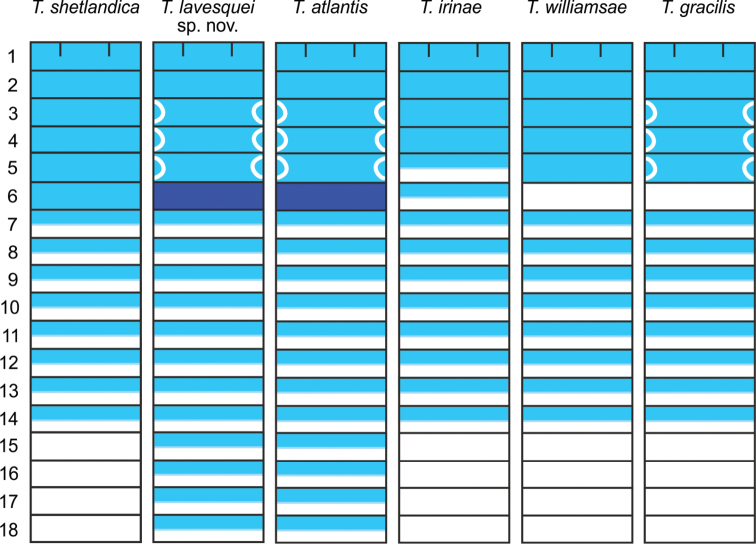
Body MG staining patterns in ventral view of *Terebellides* species. *Terebellidesshetlandica* Parapar, Moreira & O’Reilly, 2016, *Terebellideslavesquei* sp. nov., *Terebellidesatlantis* Williams, 1984, *Terebellidesirinae* Gagaev, 2009, *Terebellideswilliamsae* Jirkov, 1989 and *Terebellidesgracilis* Malm, 1874. Segments indicated in Arabic numbers.

##### Remarks.

*Terebellidesshetlandica* is a small species, reaching up to 16 mm length and is characterised by having branchiae of type 3 and long filaments in ventral branchial lobes, thoracic uncini of type 4, abdominal uncini of type 2 and lacking papillae on margins of branchial lamellae (Table 1). Parapar et al. (2016a) pointed out that *T.atlantis* is the most similar species to *T.shetlandica*; this is confirmed here according to molecular analyses and morphological examination. Both species are small sized (length: *T.shetlandica*, 5–16 mm; *T.atlantis*, 10–16 mm) and have branchiae of type 3, with free branchial lobes. However, the branchiae of *T.shetlandica* have a high number (22–26) of tightly packed branchial lamellae, all lobes are similar in shape and length and ventral ones bear long filaments whereas *T.atlantis* has a fewer number of branchiae (10–11), lamellae are not packed, lobes differ in shape and size and ventral lobes bear shorter filaments. Furthermore, the range of abdominal chaetigers number is higher in *T.shetlandica* than in *T.atlantis* (25–34 vs. 23–28 respectively).

#### ﻿ Terebellides lavesquei sp. nov.

Taxon classificationAnimaliaTerebellidaTrichobranchidae

106D1741-571D-5BC4-A015-16381CF49762

https://zoobank.org/2D993190-50A3-42C0-B11E-508EA59276B6

Figs 2C , 3B , 4B , 6 , 7 , 9 , 10B , 11 , 12 , Table 1 , Suppl. materials 1 , 2

Species 5 – Nygren et al. 2018: 18–22, figs 6, 10.

##### Material examined.

Type material. ***Holotype***: ZMBN116322. ***Paratypes*** (16 specimens): Skagerrak (GNM15112); Norwegian coast (NTNU–VM61386, NTNU–VM61387, NTNU–VM68252, ZMBN116319, ZMBN116320, ZMBN116321, ZMBN116323, ZMBN116324, ZMBN116325, ZMBN116326, ZMBN116327, ZMBN116328, ZMBN116329, ZMBN116330, ZMBN116331, ZMBN116332).

##### Holotype.

Complete specimen, 34.0 mm long and 2.0 mm wide (Fig. 4B); female with oocytes in body cavity.

##### GenBank accession numbers of material examined (COI).

MG025054, MG025055, MG025056, MG025057, MG025058, MG025059, MG025060, MG025061, MG025062, MG025063, MG025064, MG025065, MG025066, MG025067, MG025068, MG025069, MG025070.

##### Diagnostic features of type material.

Complete individuals ranging from 5.0–35.0 mm in length (Fig. 9). Branchial dorsal lobes lamellae provided with well-developed papillary projections and branchial ventral lobes provided with long filaments, ranging from 125.0–250.0 µm in length (Fig. 6A). Between 17–42 lamellae on dorsal lobes (Fig. 6A, B). Ciliary rows present on lamellae inner face (Fig. 6B, C). Ventral branchial lobes hidden in between dorsal ones but sometimes discernible below (Fig. 3B). Lateral lappets present on T C1–4; dorsal projection of thoracic notopodia on TC 2–4 (Fig. 3B). Geniculate chaetae in TC 5, acutely bent, with well-defined capitium (Fig. 7A). Ciliated papilla dorsal to thoracic notopodia not observed. From TC 7, neuropodia with one or two rows of type 3 thoracic uncini per torus, with rostrum/capitium length ratio of ~ 2:1 and capitium with a first row of four or five medium-sized teeth, followed by several smaller teeth (Fig. 7B, C). Abdomen with 30–31 pairs of neuropodia with type 2 uncini (Fig. 7D). Copepods observed attached to body dorsal surface in one specimen (Fig. 6D, E).

##### Colour pattern.

MG staining pattern characterised by compact green colourant in SG 1–6, J-shaped glandular region in SG 3–5 and striped pattern in SG 7–14 (Fig. 12). Similar to pattern 9.

##### Nucleotide diagnostic features.

All sequences of *Terebellideslavesquei* sp. nov. share and are distinguished from other available *Terebellides* sequences in unique combinations of nucleotides (underlined) at the given position of our alignment: 78–99: TCAACCCGGTGCTTACCTCGGT, 156–174: TTTAGTTATGCCAGTCTTC, 261–264: GTTA, 270–279: TCCAGCACTT, 315–336: AGTTGGGACCGGTTGAACCGTT, 351–369: AGACAATATAGCTCATGCG, 405–411: CTTGGCT, 426–447: CCTAGGATCAATTAACTTTATC, 459–483: CAACATACGCTGAAAAGGTTTACGA, 510–525: GTCCGCGGTTATCACA, 534–558: ACTTCTTTTATCCCTTCCAGTCTTG, 573–580: CATGCTTC, 606–627: CTTTTTCGACCCAGCTGGTGGG.

##### Type locality.

Hordaland, Lysefjord (Norway), 60°07'N, 05°04'E; 119 m deep.

##### Distribution and bathymetry.

Norwegian coast and shelf, Skagerrak; 115–534 m deep; ~ 50% of specimens collected at depths above 200 m (Figs 10B, 11, Suppl. material 1).

##### Etymology.

This species is dedicated to Nicolas Lavesque, Station Marine d’Arcachon, CNRS (France) for his remarkable recent contributions to the diversity of Terebellidae and Trichobranchidae in Atlantic waters.

##### Remarks.

*Terebellideslavesquei* sp. nov. is a medium-sized species, reaching up to 35 mm in length. It is characterised by the lack of papillae on margins of branchial lamellae and by having branchiae of type 2, filaments on ventral branchial lobes, thoracic uncini of type 3 and abdominal uncini of type 2 (Table 1). *Terebellideslavesquei* sp. nov. is genetically close to *T.shetlandica* and *T.atlantis* but mostly differs from them regarding branchiae features (Table 1). Lobes are partially fused and have many tightly packed lamellae (17–42) in comparison with these species. *Terebellideslavesquei* sp. nov. is also similar to *Terebellidesparapari* Lavesque, Hutchings, Daffe, Nygren & Londoño-Mesa, 2019 in having filaments in ventral branchial lobes and the presence of glandular regions, but they differ in the branchial morphology, with lobes fused ca. half of their length in *T.lavesquei* sp. nov. and fused only at the base in *T.parapari*. They also differ in TC 1 notochaetae length, being all similar in *T.lavesquei* sp. nov. but longer than those in following chaetigers in *T.parapari*.

Branchial shape of *T.lavesquei* sp. nov. is similar to that of *Terebellidesnarribri* Hutchings & Peart, 2000, because both lobes are fused to each other for ca. half their length and have a high number of tightly packed lamellae. However, *T.narribri* have thoracic uncini of type 1 whereas *T.lavesquei* sp. nov. have thoracic uncini of type 3. Furthermore, *T.lavesquei* sp. nov. and *T.shetlandica* seem to have a more restricted bathymetric distribution in shallow waters (down to 534 and 375 m, respectively) whereas *T.atlantis* reaches depths of 2750 m (see below).

#### ﻿ Terebellides atlantis

Taxon classificationAnimaliaTerebellidaTrichobranchidae

Williams, 1984

03F3107D-0D36-5CBF-8734-7954AD5ED893

Figs 3C , 8 , 9 , 10C , 11 , 12 , Table 1 , Suppl. materials 1 , 2

Terebellides atlantis Williams, 1984: 121–123, fig. 4, table 1.
Species 16 – Nygren et al. 2018: 18–22, figs 6, 10.

##### Material examined.

15 specimens (Suppl. material 1), Barents Sea (ZMBN116454, ZMBN116455, ZMBN116458, ZMBN116459, ZMBN116460, ZMBN116462, ZMBN116463, ZMBN116465, ZMBN116467, ZMBN116468, ZMBN116470, ZMBN116471, ZMBN116472, ZMBN116474); Norwegian coast (ZMBN116476).

##### GenBank accession numbers of material examined (COI).

MG025258, MG025259, MG025260, MG025261, MG025262, MG025263, MG025264, MG025265, MG025266, MG025267, MG025268, MG025269, MG025270, MG025271, MG025272, MG025273, MG025274, MG025275, MG025276, MG025277, MG025278, MG025279, MG025280, MG025281, MG025282, MG025283, MG025284, MG025285, MG025286, MG025287, MG025288, MG025289, MG025290, MG025291, MG025292, MG025293, MG025294, MG025295, MG025296, MG025297, MG025298, MG025299, MG025300, MG025301, MG025302, MG025303, MG025304, MG025305, MG025306, MG025307, MG025308, MG025309, MG025310, MG025311, MG025312.

##### Diagnostic features of studied material.

Complete individuals ranging from 10.0–16.0 mm in length (Fig. 9). Branchial dorsal lobes lamellae provided with well-developed papillary projections and branchial ventral lobes (Fig. 8A, B) provided with long filaments (sometimes broken), 175.0 µm in length. Between 10–11 lamellae on dorsal lobes. Lateral lappets present on TC 1–4; dorsal projection of thoracic notopodia on TC 2–4 (Fig. 8A). Geniculate chaetae in TC 5, acutely bent, with well-defined capitium (Fig. 8C). Ciliated papilla dorsal to thoracic notopodia not observed. From TC 7, neuropodia with one row of type 3 thoracic uncini per torus, with rostrum/capitium length ratio of ~ 2:1 and capitium with a first row of three or four medium-sized teeth, followed by several smaller teeth (Fig. 8D). Abdomen with 23–28 pairs of neuropodia with type 2 uncini (Fig. 8E, F).

##### Colour pattern.

MG staining pattern characterised by compact green colourant in SG 1–6, J-shaped glandular region in SG 3–5 and striped pattern in SG 7–14 (Fig. 12). Similar to pattern 9.

##### Nucleotide diagnostic features.

All sequences of *Terebellidesatlantis* share and are distinguished from other available *Terebellides* sequences in unique combinations of nucleotides (underlined) at the given position of our alignment: 60–84: TATTCGTATTGAGCTAGGGCAACCT, 132–150: ACATGCATTTTTAATAATC, 171–189: TTTTATTGGTGGATTTGGT, 213–231: GGGAGCTCCTGATATAGCC, 264–294: ACTACCACCAGCCTTAATCTTATTAGTAAGC, 345–363: ATTATCTGATAATATGGCT, 384–399: CCTTGCTATTTTTTCA, 477–484: GCTACGAC, 549–573: TCCAGTCTTAGCTGGTGCAATCACT, 558–591: CCGT, 615–630: TCCAGCTGGTGGTGGT.

##### Type locality.

Atlantic Ocean, off New England, 39°56.5'N, 70°39.9'W (Williams 1984).

##### Distribution and bathymetry.

Barents Sea, Greenland Sea, South Iceland, Norwegian coast and shelf; 219–2750 m deep (Figs 10C, 11, Suppl. material 1).

##### Remarks.

*Terebellidesatlantis* is a small species, reaching up to 16 mm in length. It is characterised by the lack of papillae on margins of branchial lamellae, and by having branchiae of type 3 and filaments in ventral branchial lobes, thoracic uncini of type 3 and abdominal uncini of type 2 (Table 1). The most similar species to *T.atlantis* are *T.shetlandica* and *T.lavesquei* sp. nov. but *T.atlantis* differs from the latter in the size and type of branchiae (see remarks for *T.lavesquei* sp. nov. above). Branchial lobes are often missing as previously highlighted by Parapar et al. (2011). Finally, *T.atlantis* has the widest geographical distribution and depth range (219 to 2750 m) among Group B species.

#### ﻿ Terebellides

Taxon classificationAnimaliaTerebellidaTrichobranchidae

Group C (sensu Nygren et al. 2018)

B06F73F7-8E65-529D-8ACF-9EC4FB5A480F

**Figure 13.**
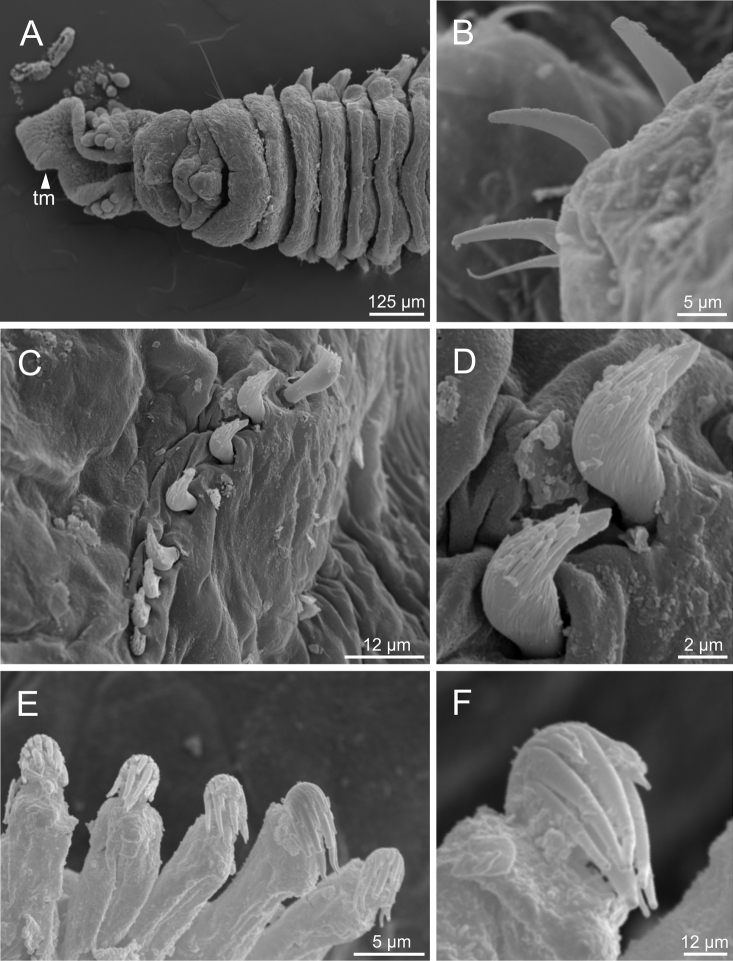
*Terebellidesirinae* Gagaev, 2009 (species 24; non-type specimen, ZMBN116501), SEM micrographs. **A** anterior end, ventral view **B**TC 6 (TU1), geniculate chaetae **C** row of thoracic uncini **D** thoracic uncini **E** abdominal uncini **F** abdominal uncinus, detail. Abbreviations: tc – thoracic chaetiger; tm – tentacular membrane; tu – thoracic unciniger.

Figs 3 , 4 , 9 , 10 , 11 , 12 , 13

##### Description.

The morphological features of the examined species in Group C in this paper (clade 24) are itemised below. Some of these are also shared by Groups A, B, and D as defined in Nygren et al. (2018) (see Remarks below). Clade 25 will be studied elsewhere; formal description of clade 22 will wait until more material is available.

***Body appearance*.** Incomplete individuals ranging from 10.0–17.0 mm in length. Body tapering posteriorly with segments increasingly shorter and crowded towards pygidium. Prostomium compact; large tentacular membrane surrounding mouth (Fig. 3D), with typical buccal tentacles with expanded tips (Fig. 3D). SG 1 as an expanded structure below tentacular membrane in a lower lip (Fig. 3D).

***Branchiae*.** Branchiae arising as single structure from SG 3, with a single stalked mid-dorsal stem, lobes not fused (Fig. 4C). Dorsal lobes ending posteriorly in short terminal papilla (Fig. 3D) and ventral lobes ones ending in long filaments. Anterior projection of dorsal lobes (fifth lobe) present. Posterior end of dorsal lobes reaching TC 4. Ciliary rows of cilia and ciliary tufts in inner face of branchial lamellae not observed. Ciliary papillae absent in branchial lamellae margin.

***Thorax*.** Eighteen pairs of notopodia (SG 3–20) (Fig. 3D), those of TC 1 approximately as long as subsequent ones (Fig. 4C). Lateral lappets and dorsal projections of notopodia in anterior thoracic chaetigers with different degree of development depending on size and preservation conditions, but both more conspicuous on TC 1–5. All notochaetae as simple capillaries. Size of notochaetae of TC 1 similar to subsequent ones. Neuropodia as sessile pinnules from TC 6 to body end, with uncini in single rows, from TC 7 throughout. Neuropodia on TC 6, provided with several sharply bent, acute-tipped, geniculate chaetae (Fig. 13B) with minute teeth forming a capitium only visible with SEM (Fig. 13B). From TC 7, neuropodia with one row of uncini per torus (Fig. 13C), with long shafted denticulate hooks, with large main fang (rostrum) longer than upper crest of teeth (capitium), rostrum/capitium length ratio of ~ 2:1, capitium composed by several teeth above main fang of decreasing length (Fig. 13D).

***Abdomen and pygidium*.** Approximately half as long as thorax and progressively thinner (Fig. 3D). Neuropodia ranging from 18–20 chaetigers and forming erect pinnules with several uncini per torus, number depending on specimen size. Uncini provided with several teeth above rostrum surmounted by a capitium composed of several teeth of decreasing length (Fig. 13E, F). Pygidium blunt, as funnel-like depression.

***Colour pattern*.** Colour in preserved specimens whitish (Fig. 3D). MG staining pattern characterised by compact green colourant in SG 1–4, then turning into striped pattern in SG 5–14 and fading in following segments (Fig. 12).

##### Remarks.

Among the above-mentioned characters, branchial features might serve to distinguish most of Group C species from those of Groups A, B and D. Those include branchial lobes size, presence of filaments in ventral ones and lobes which are not fused. Other taxa such as *Terebellidesmira* Schüller & Hutchings, 2013 and *T.rigel* Schüller & Hutchings, 2013 also bear branchiae with similar shape (Parapar et al. 2016a).

#### ﻿ Terebellides irinae

Taxon classificationAnimaliaTerebellidaTrichobranchidae

Gagaev, 2009

A0820D23-7F6D-526D-8A6D-482AA90CF569

Figs 3D , 4C , 9 , 10D , 11 , 12 , 13 , Table 1 , Suppl. materials 1 , 2

Terebellides irinae Gagaev, 2009: 474–478.
Species 24 – Nygren et al. 2018: 18–22, figs 6, 10.

##### Material examined.

6 specimens (Suppl. material 1), Arctic Ocean (ZMBN116496, ZMBN116497, ZMBN116498, ZMBN116499, ZMBN116500, ZMBN116501).

##### GenBank accession numbers of material examined (COI).

MG025340, MG025341, MG025342, MG025343, MG025344.

##### Diagnostic features of studied material.

Incomplete individuals ranging from 10.0–17.0 mm in length (Fig. 9). Branchial dorsal lobes provided with filaments, 75.0 µm in length (Fig. 3D) and branchial ventral lobes reduced, distinctly smaller than dorsal ones (Fig. 4C). Dorsal lobes provided with seven lamellae (Fig. 4C). Lateral lappets present on TC 1–4; dorsal projection of thoracic notopodia on TC 2–5 (Fig. 3D). Geniculate chaetae in TC 5, acutely bent and provided with hardly distinguishable capitium (Fig. 13B). Ciliated papilla dorsal to thoracic notopodia not observed. From TC 7, neuropodia with one row of type 3 thoracic uncini per torus, with rostrum/capitium length ratio of ~ 2:1 and capitium with a first row of four or five medium-sized teeth, followed by several smaller teeth (Fig. 13C, D). Abdomen with at least 20 pairs of neuropodia with type 2 uncini (Fig. 13E, F).

##### Colour pattern.

MG staining pattern characterised by compact green colourant in SG 1–4, then turning into striped pattern in SG 5–14 and fading in following segments (Fig. 12). Similar to pattern 1.

##### Nucleotide diagnostic features.

All sequences of *Terebellidesirinae* share and are distinguished from other available *Terebellides* sequences in unique combinations of nucleotides (underlined) at the given position of our alignment: 177–204: CGGGGGGTTTGGAAACTGGTTAATCCCC, 213–225: TGGGGCCCCAGAC, 249–258: CATAAGGTTC, 273–303: GGCCCTCATCCTACTAGTCAGCTCAGCTGCT, 305–321: GGCTGGT, 327–336: ATGAACTGTA, 342–372: ACCACTTTCAGACAACATCGCTCATGCCGGA, 381–399: AGATCTAGCAATTTTCTCA, 426: CCTAGGTTCTATTAACTTCATCACAACAGTC, 483–499: TCTAGAACGAATCCCAC, 535–573: TTATTACTATCACTACCAGTGCTAGCCGGAGCTATTACC, 594–612: CATTAACACATCATTCTTC, 618–636: AGCCGGTGGTGGTGATCCT.

##### Type locality.

Arctic Ocean, 73°04'N, 157°12'W (Gagaev 2009).

##### Distribution and bathymetry.

Arctic Ocean; 4038–4380 m deep (Figs 10D, 11, Suppl. material 1).

##### Remarks.

*Terebellidesirinae* is a small species, reaching up to 17 mm in length and is characterised by the lack of papillae on margins of branchial lamellae, and by having branchiae of type 4, filaments in ventral branchial lobes, thoracic uncini of type 3 and abdominal uncini of type 2 (Table 1). Jirkov and Leontovich (2013) proposed *T.irinae* as synonym of *T.stroemii* because it fit within the variability of the latter. However, Parapar and Hutchings (2014) redescribed *T.stroemii* designating a neotype and *T.irinae* not fit in this concept. Later, Nygren et al. (2018) recognised *T.irinae* as different from *T.stroemii* after molecular analyses and pointed out that *T.irinae* is the only species present in the Arctic Ocean at depths below 4000 m (Fig. 11). Furthermore, *T.irinae* is the only species in Northeast Atlantic Ocean bearing branchiae of type 4 and therefore is also considered as a valid species in this work. Other taxa from elsewhere such as *T.mira* and *T.rigel* also bear the same branchial type, these two species have branchial lobes free from each other with few numbers of not packed lamellae and ventral lobes are also distinctly smaller than the dorsal ones.

#### ﻿ Terebellides

Taxon classificationAnimaliaTerebellidaTrichobranchidae

Group D (sensu Nygren et al. 2018)

9D7B9BB3-09D0-5C1A-B67A-DD154ED33036

Figs 2 , 3 , 4 , 9 , 10 , 11 , 12 , 13 , 14 , 15 , 16 , 17 , 18

##### Description.

The morphological features shared by all examined species in Group D in this paper (clades 2 and 3) are itemized below. Some of these are also shared by Groups A, B, and C as defined in Nygren et al. (2018) (see Remarks below). Clade 15 will be studied elsewhere.

***Body appearance*.** Complete individuals ranging from 5.0–34.0 mm in length. Body tapering posteriorly with segments increasingly shorter and crowded towards pygidium. Prostomium compact; large tentacular membrane surrounding mouth (Figs 2D–F, 3E, F), with typical buccal tentacles with expanded tips (Figs 2E, F, 3E, F). SG 1 as an expanded structure below tentacular membrane in a lower lip (Figs 2D, E, 3E, F).

**Figure 14.**
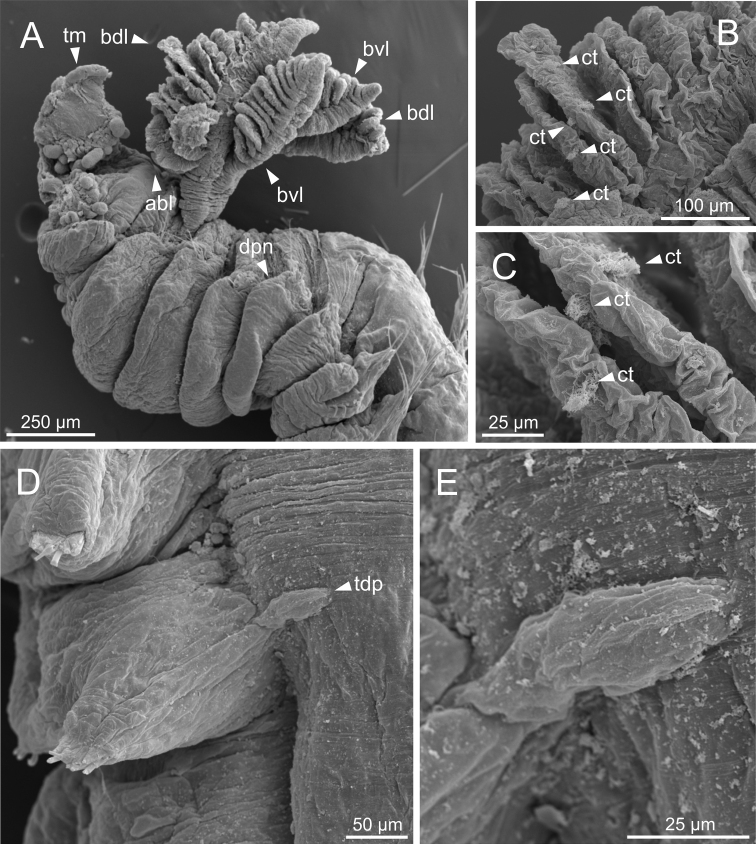
*Terebellideswilliamsae* Jirkov, 1989 (species 2; non-type specimens, ZMBN116249 and ZMBN116251), SEM micrographs **A** anterior end, left lateral view **B** branchial lamellae, detail **C** ciliary tufts, detail **D**TC and thoracic dorsal papilla **E** thoracic dorsal papilla, detail. Abbreviations: abl – anterior branchial lobe; bdl – branchial dorsal lobe; bvl – branchial ventral lobe; ct – ciliary tuft; dpn – dorsal projection of notopodium; TC – thoracic chaetiger; tdp – thoracic dorsal papilla; tm – tentacular membrane.

***Branchiae*.** Branchiae arising as single structure from SG 3, with a single stalked mid-dorsal stem (Figs 2D, E, 3E, F), one pair of dorsal (upper) partially fused lobes (Figs 2D, E, 3E, F), and a pair of shorter ventral (lower) lobes (Fig. 3E, F) obscured or not by dorsal ones (Figs 2D–F, 3E, F). Dorsal lobes ending posteriorly in short terminal papilla (Fig. 3E, F) and ventral lobes ending in long filaments. Anterior projection of dorsal lobes (fifth lobe) present (Fig. 2D–F). Posterior end of dorsal lobes reaching TC 4–5 (Figs 2D–F, 3E, F). Branchial lamellae provided with several parallel rows of cilia and ciliary tufts present in inner face (Figs 14B, C, 16B, C, 17B). Ciliary papillae absent on the margin of branchial lamellae.

**Figure 15.**
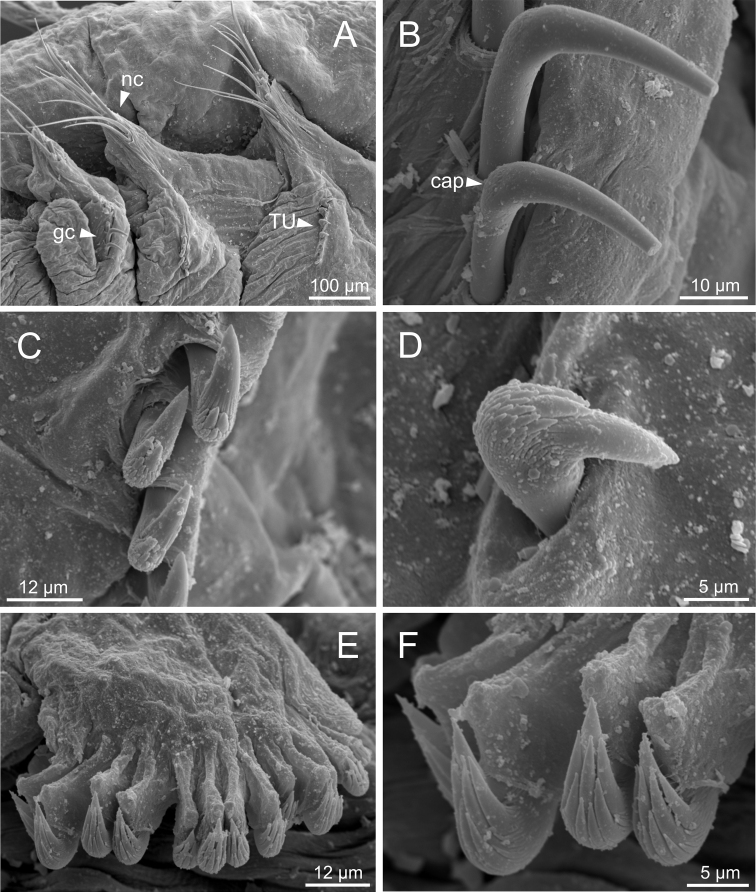
*Terebellideswilliamsae* Jirkov, 1989 (species 2; non-type specimen, ZMBN116249), SEM micrographs **A**TC 5–7, lateral view **B**TC 6 (TU1), geniculate chaetae **C** row of thoracic uncini **D** thoracic uncinus **E** abdominal neuropodium **F** abdominal uncini. Abbreviations: cap – capitium; gc – geniculate chaetae; nc – notochaetae; TC – thoracic chaetiger; TU – thoracic unciniger.

***Thorax*.** Eighteen pairs of notopodia (SG 3–20) (Fig. 2D, E), those of TC 1 approximately as long as subsequent ones (Fig. 2D, E). Lateral lappets and dorsal projections of notopodia in anterior thoracic chaetigers with different degree of development depending on size and preservation conditions, but both more conspicuous on TC 1–5 (Figs 2D–F, 3E, F). White ventral colouration present on TC 1–4 (Figs 2D, 3E) or only on TC 4 (Figs 2E, F, 3F). All notochaetae as simple capillaries (Fig. 15A). Size of notochaetae of TC 1 similar to subsequent ones. Neuropodia as sessile pinnules from TC 6 to body end, with uncini in single or double rows, from TC 7 throughout. Neuropodia on TC 6, provided with several sharply bent, acute-tipped, geniculate chaetae (Figs 15B, 18A) with minute teeth forming a capitium only visible with SEM (Fig. 18A, B). From TC 7, neuropodia with one row of uncini per torus (Figs 15C, 18C), with long shafted denticulate hooks, with large main fang (rostrum) longer than upper crest of teeth (capitium), rostrum/capitium length ratio of ~ 2:1, capitium composed by several teeth above main fang of decreasing length (Figs 15D, 18D).

**Figure 16.**
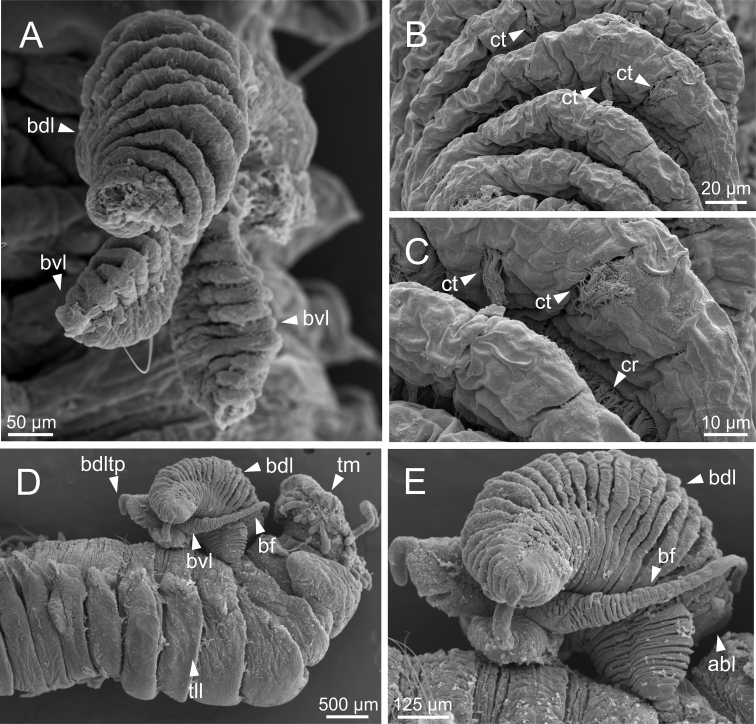
*Terebellidesgracilis* Malm, 1874 (species 3; non-type specimens, GNM15110 and ZMBN116313), SEM micrographs **A** branchiae, dorsal view **B** branchial lamellae, detail **C** ciliary tufts, detail **D** anterior end, right lateral view **E** branchiae, lateral view. Abbreviations: abl – anterior branchial lobe; bdl – branchial dorsal lobe; bdltp – branchial dorsal lobe terminal papilla; bf – filament; bvl – branchial ventral lobe; cr – ciliary row; ct – ciliary tuft; tll – thoracic lateral lobes; tm – tentacular membrane.

**Figure 17.**
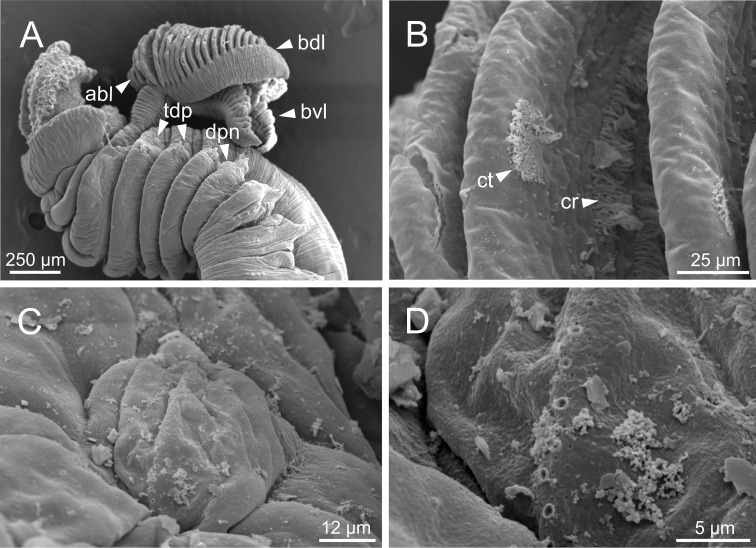
*Terebellidesgracilis* Malm, 1874 (species 3; non-type specimen, ZMBN116282), SEM micrographs **A** anterior end, left lateral view **B** ciliary tufts and ciliary row, detail **C** dorsal projection of notopodium **D** pores of dorsal projection of notopodium, detail. Abbreviations: abl – anterior branchial lobe; bdl – branchial dorsal lobe; bvl – branchial ventral lobe; cr – ciliary row; ct – ciliary tuft; dpn – dorsal projection of notopodium; tdp – thoracic dorsal papilla.

**Figure 18.**
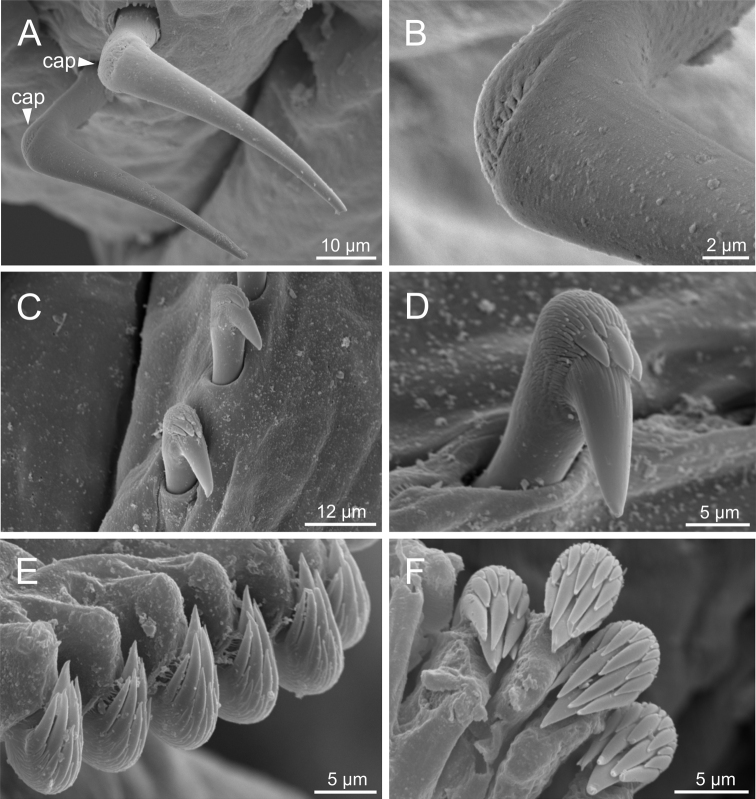
*Terebellidesgracilis* Malm, 1874 (species 3; non-type specimens, ZMBN116282 and ZMBN116313), SEM micrographs **A**TC 6 (TU1), geniculate chaetae **B** capitium of geniculate chaeta, detail **C** thoracic uncini **D** thoracic uncinus **E** abdominal neuropodium **F** abdominal uncini. Abbreviations: cap – capitium; TC – thoracic chaetiger; TU – thoracic unciniger.

***Abdomen and pygidium*.** Approximately half as long as thorax and progressively thinner (Fig. 2D, E). Neuropodia ranging from 18–44 chaetigers and forming erect pinnules (Figs 15E, 18E) with several uncini per torus, number depending on specimen size. Uncini provided with several teeth above rostrum surmounted by a capitium composed of several teeth of decreasing length (Figs 15F, 18F). Pygidium blunt, as funnel-like depression.

***Colour pattern*.** Colour in preserved specimens whitish or pale brown (Fig. 3E, F). MG staining pattern characterised by compact green colourant in SG 1–5 and SG 7–13, SG 6 white and SG 14 striped (Fig. 12).

##### Remarks.

Among the aforementioned characters, the white ventral colouration in anterior thoracic chaetigers may be a useful character to distinguish Group D species from those of Groups A–C. Other taxa described or reported worldwide showing this colouration pattern are *Terebellidesdistincta* Williams, 1984 and *T.ceneresi* Lavesque, Hutchings, Daffe, Nygren & Londoño-Mesa, 2019.

#### ﻿ Terebellides williamsae

Taxon classificationAnimaliaTerebellidaTrichobranchidae

Jirkov, 1989

8176354B-1320-50CD-A3DF-013FC3224B8F

Figs 2D , 3E , 9 , 10E , 11 , 12 , 14 , 15 , Table 1 , Suppl. materials 1 , 2

Terebellides williamsae Jirkov, 1989: 124.
Species 2 – Nygren et al. 2018: 18–22, figs 6, 10.

##### Material examined.

20 specimens (Suppl. material 1), Skagerrak (GNM14639, GNM15107, GNM15108); Barents Sea (ZMBN116246, ZMBN116247, ZMBN116248, ZMBN116249, ZMBN116251, ZMBN116252, ZMBN116253, ZMBN116254, ZMBN116255, ZMBN116257, ZMBN116260, ZMBN116262, ZMBN116263, ZMBN116266, ZMBN116269, ZMBN116270, ZMBN116271).

##### GenBank accession numbers of material examined (COI).

MG024957, MG024958, MG024959, MG024960, MG024961, MG024962, MG024963, MG024964, MG024965, MG024966, MG024967, MG024968, MG024969, MG024970, MG024971, MG024972, MG024973, MG024974, MG024975, MG024976, MG024977, MG024978, MG024979, MG024980, MG024981, MG024982, MG024983, MG024984, MG024985, MG024986, MG024987, MG024988.

##### Diagnostic features of studied material.

Complete individuals ranging from 9.0–34.0 mm in length (Fig. 9). Branchial dorsal lobes lamellae provided with well-developed papillary projections and branchial ventral lobes provided with short posterior filaments, 50.0 µm in length (Figs 3E, 14A). Between 16–18 lamellae on dorsal lobes (Fig. 14A, B). Ciliary tufts present in inner face of lamellae (Fig. 14B, C). Ventral branchial lobes hidden in between dorsal ones but sometimes discernible below (Fig. 14A). Lateral lappets present on TC 1–4; dorsal projection of thoracic notopodia on TC 2–4 (Fig. 14A). White ventral colouration present on TC 1–4 (Figs 2D, 3E). Geniculate chaetae in TC 5, acutely bent, with well-marked capitium (Fig. 15B). Ciliated papilla dorsal to thoracic notopodia observed in TC 7 (Fig. 14D, E). From TC 7, neuropodia with one row of type 1 thoracic uncini per torus, with rostrum/capitium length ratio of ~ 2:1 and capitium with a first row of two or three large teeth, followed by many smaller teeth (Fig. 15C, D). Abdomen with 38–44 pairs of neuropodia with type 1A uncini (Fig. 15E, F).

##### Colour pattern.

MG staining pattern characterised by compact green colourant in SG 1–5 and SG 7–13, SG 6 white and SG 14 striped, J-shaped glandular regions in SG 3–5 (Fig. 12). Similar to pattern 2.

##### Nucleotide diagnostic features.

All sequences of *Terebellideswilliamsae* share and are distinguished from other available *Terebellides* sequences in unique combinations of nucleotides (underlined) at the given position of our alignment: 59–62: TATC, 75–96: TGGACAACCTGGGGCATTCCTG, 132–144: TCATGCTTTTTTA, 153–157: TTTCC, 216–234: TGCTCCTGATATAGCTTTC, 264–277: CCTCCCTCCAGCTT, 315–318: GGTT, 327–342: CTGAACAGTATACCCC, 381–399: AGATTTGGCTATTTTTTCT, 414–432: TATCTCCTCTATTCTTGGC, 450–454: TACA, 515–529: AAAAATCACTACCA, 543–573: TTCACTTCCTGTATTAGCAGGAGCTATTACA, 600–609: CACTTCCTTT, 630–640: CGACCCAATTT.

##### Type locality.

Barents Sea, Norway, 74°30'N, 28°00'E (Jirkov 1989).

##### Distribution and bathymetry.

Barents Sea, Greenland Sea, Norwegian coast and shelf, Skagerrak; at depths of 178–612 m but most of the specimens (97%) were collected above 200 m (Figs 10E, 11, Suppl. material 1).

##### Remarks.

*Terebellideswilliamsae* is a medium-sized species, reaching up to 34 mm in length; it is characterised by the lack of papillae on margins of branchial lamellae and by having branchiae of type 2 and posterior filaments in ventral branchial lobes, thoracic uncini of type 1 and abdominal uncini of type 1A (Table 1). All these features are shared with *T.gracilis*; in fact, Parapar et al. (2011) suggested this species as a synonym to *T.gracilis* after examining specimens from Iceland. Nygren et al. (2018) pointed out that there were no morphological differences between both species, but their molecular analyses indicate that specimens from the Barents Sea (“Species 2”) would correspond to *T.williamsae*. Nygren et al. (2018) suggested therefore that *T.williamsae* might be a valid species and different from *T.gracilis* (“Species 3”, see below). Here, examination of specimens of *T.williamsae* show that they differ from *T.gracilis* in the number of chaetigers with white ventral colouration, i.e., in *T.williamsae* white colouration is present in TC 1–4 while in *T.gracilis* it is only present on TC 4.

#### ﻿ Terebellides gracilis

Taxon classificationAnimaliaTerebellidaTrichobranchidae

Malm, 1874

96856AFC-136E-5E9E-BFD7-E498F5D59E97

Figs 2E, F , 3F , 4D , 9 , 10F , 11 , 12 , 16 , 17 , 18 , Table 1 , Suppl. materials 1 , 2

Terebellides gracilis Malm, 1874: 67–105, p. 100.
Species 3 – Nygren et al. 2018: 18–22, figs 6, 10.

##### Material examined.

20 specimens (Suppl. material 1), Skagerrak (GNM15110, GNM15111); Norwegian coast (ZMBN116276, ZMBN116278, ZMBN116282, ZMBN116283, ZMBN116284, ZMBN116285, ZMBN116287, ZMBN116289, ZMBN116293, ZMBN116295, ZMBN116297, ZMBN116298, ZMBN116301, ZMBN116306, ZMBN116307, ZMBN116309, ZMBN116310, 116313).

##### GenBank accession numbers of material examined (COI).

MG024583, MG024584, MG024585, MG024586, MG024587, MG024588, MG024589, MG024590, MG024591, MG024592, MG024593, MG024594, MG024595, MG024596, MG024597, MG024598, MG024599, MG024600, MG024601, MG024602, MG024603, MG024604, MG024605, MG024606, MG024607, MG024608, MG024609, MG024610, MG024611, MG024612, MG024613, MG024614, MG024615, MG024616, MG024617, MG024618, MG024619, MG024620, MG024621, MG024622, MG024623, MG024624, MG024625, MG024626, MG024627, MG024628, MG024629, MG024630, MG024631, MG024632, MG024633, MG024634, MG024635, MG024636, MG024637.

##### Diagnostic features of studied material.

Complete individuals ranging from 5.0–29.0 mm in length (Fig. 9). Branchial dorsal lobes lamellae provided with well-developed papillary projections and branchial ventral lobes provided with long posterior filaments, ranging from 125.0–175.0 µm in length (Fig. 16D, E). Between 23–32 lamellae on dorsal lobes (Figs 4C, 16A, D, E, 17A). Ciliary rows and ciliary tufts on inner branchial lamellae present (Figs 16B, C, 17B). Ventral branchial lobes hidden in between dorsal ones but sometimes discernible below (Figs 16A, D, 17A). Lateral lappets present on TC 1–4; dorsal projection of thoracic notopodia on TC 1–5 (Fig. 16D). White ventral colouration presents only on TC 4 (Figs 2E, F, 3F). Geniculate chaetae present in TC 5, acutely bent, with marked capitium (Fig. 18A, B). Ciliated papilla dorsal to thoracic notopodia observed in TC 2–4 (Fig. 17A, C, D). From TC 7, neuropodia with one row of type 1 thoracic uncini per torus, with rostrum/capitium length ratio of ~ 2:1 and capitium with a first row of two or three large teeth, followed by many smaller teeth (Fig. 18C, D). Abdomen with 34–41 pairs of neuropodia with type 1A uncini (Fig. 18E, F).

##### Colour pattern.

MG staining characterised by compact green colourant in SG 1–5 and SG 7–13, SG 6 white and SG 14 striped (Fig. 12). Similar to pattern 2.

##### Nucleotide diagnostic features.

All sequences of *Terebellidesgracilis* share and are distinguished from other available *Terebellides* sequences in unique combinations of nucleotides (underlined) at the given position of our alignment: 39–63: TGGTACTTCAATAAGACTTCTTATC, 84–96: TGGGGCATTCCTG, 111–132: TTATAACACAATTGTTACTGCT, 138–157: TTTTTTAATAATTTTTTTCC, 216–234: TGCTCCTGATATAGCTTTC, 264–277: CCTCCCTCCAGCTT, 315–327: AGCTGGGACAGGT, 333–351: AGTCTACCCTCCTTTATCT, 381–399: AGATTTGGCTATTTTTTCT, 414–432: TATCTCCTCTATTCTTGGC¸ 450–545: TACA, 516–529: AAAAATCACTACCA, 543–552: TTCACTTCCT, 600–609: CACTTCCTTT, 630–640: CGACCCAATTT.

##### Type locality.

Atlantic Ocean, Norway (Malm 1874).

##### Distribution and bathymetry.

South Iceland, Norwegian coast and shelf, Skagerrak; 237–1268 m deep (Figs 10F, 11, Suppl. material 1).

##### Remarks.

*Terebellidesgracilis* is a medium-sized species, reaching up to 29 mm in length and is characterised by the lack of papillae on margins of branchial lamellae, having branchiae of type 2 and filaments in ventral branchial lobes, presence of thoracic uncini of type 1 and abdominal uncini of type 1A (Table 1). As stated above, these features are shared with *T.williamsae* but both species differ in the pattern of white ventral thoracic colouration. Besides, they show a MG pattern close to type 2 but only *T.gracilis* showed J-shaped glandular regions in SG 3–5 as observed in the specimens studied here. *Terebellidesgracilis* has apparently a more restricted geographical distribution than *T.williamsae* but reaching deeper depths (down to 1268 m).

###### ﻿Key to Northeast Atlantic Ocean species of *Terebellides*

The following key of European species of *Terebellides* is based on those by Lavesque et al. (2019) and Parapar et al. (2020a) but has been updated to include the species belonging to Groups B, C and D studied herein. The order of the presentation of the discriminating characters and the taxa has been changed to fit better with the clades recovered in the phylogenetic trees by Nygren et al. (2018) and Lavesque et al. (2019).

The characters considered were the ventral pigmentation of anterior thoracic chaetigers in live and fixed specimens, types of thoracic uncini (sensu Parapar et al. 2020b), morphology of branchiae (sensu Parapar et al. 2016a), morphology of the abdominal uncini (sensu Parapar et al. 2020a), the size of species (small species: < 20 mm in length; medium: 20–40 mm; large: > 40 mm), the presence of geniculate chaetae in TC 5–6 or only in TC 6, the presence or absence of papillae in branchial lamellae margins, the shape of glandular region in TC 3, and the presence or absence of ciliary tufts in branchial lamellae. In those cases where two species are considered as cryptic and only distinguished by molecular characters, geographic and bathymetric distribution has been provided instead.

**Table:** 

1	White ventral colouration on anterior thoracic chaetigers	**2**
–	No distinct ventral colouration on anterior thoracic chaetigers	**4**
2	Medium/large species (>20 mm in length); 5^th^ branchial lobe present; notochaetae of TC 1 similar to subsequent ones; main fang of thoracic uncini straight; thoracic uncini with capitium composed of 2–3 large teeth and subsequent ones much smaller	**3**
–	Small species (< 20 mm in length); 5^th^ branchial lobe absent; notochaetae of TC 1 absent or shorter than subsequent ones; thoracic uncini with capitium composed of 4 or 5 mid-sized teeth and following of slightly smaller teeth	***T.ceneresi* Lavesque, Hutchings, Daffe, Nygren & Londoño-Mesa, 2019**
3	White ventral colouration on TC 1 to TC 4	***T.williamsae* Jirkov, 1989**
–	White ventral colouration only on TC 4	***T.gracilis* Malm, 1874**
4	Branchial lobes all small and not fused; reduced dorsal lobes	***T.irinae* Gagaev, 2009**
–	Branchiae otherwise	**5**
5	Lower branchial lobes with posterior projections as filaments; branchiae with lobes fused ~ 50% of their length or with lobes only fused at base; small/medium species (<40 mm in length)	**6**
–	Lower branchial lobes with posterior projections; branchiae with large lobes almost completely fused; large species (> 40 mm in length)	**9**
6	Thoracic uncini with capitium composed of 5–7 small teeth, remaining ones similar in size at least in two rows	***T.shetlandica* Parapar, Moreira & O’Reilly, 2016**
–	Thoracic uncini with capitium composed of 4–5 mid-sized teeth and followed by slightly smaller teeth	**7**
7	Branchiae with lobes fused ~ 50% of their length; medium-sized species (> 20 mm in length)	***T.lavesquei* sp. nov.**
–	Branchiae with lobes only fused at base; small species (< 20 mm in length)	**8**
8	Glandular region in TC 3 present; notochaetae from TC 1 longer than subsequent ones	***T.parapari* Lavesque, Hutchings, Daffe, Nygren & Londoño-Mesa, 2019**
–	Glandular region in TC 3 not observed; all notochaetae of similar size	***T.atlantis* Williams, 1984**
9	Geniculate chaetae in TC 5 and TC 6; abdominal uncini with RvC = 1/0.7, capitium with 4–5 teeth and remaining ones smaller	***T.bigeniculatus* Parapar, Moreira & Helgason, 2011**
–	Geniculate chaetae in TC 6 only	**10**
10	Branchial lamellae margins lacking papillae	**11**
–	Branchial lamellae margins with papillae	**13**
11	Branchiae with lobes fused ~ 50% of their length	***T.gralli* Lavesque, Hutchings, Daffe, Nygren & Londoño-Mesa, 2019**
–	Branchiae with large lobes almost completely fused	**12**
12	Abdominal uncini with RvC = 1/0.7, capitium with 4–5 teeth and remaining ones smaller	***T.stroemii* Sars, 1835**
–	Abdominal uncini with RvC = 1/0.9, capitium composed of 3–5 large teeth in first row and 1–2 in a second row	***T.kongsrudi* Parapar, Capa, Nygren & Moreira, 2020** and ***T.bakkeni* Parapar, Capa, Nygren & Moreira, 2020**
13	Glandular region in TC 3 round or oval	**14**
–	Glandular region in TC 3 otherwise	**15**
14	Glandular region in TC 3 remained white with MG; branchial lamellae with rounded papillae; TC 1–3 without conspicuous dorsal projection	***T.lilasae* Lavesque, Hutchings, Daffe, Nygren & Londoño-Mesa, 2019**
–	Glandular region in TC 3 stained blue with MG; branchial lamellae with conical papillae; TC 1–3 with conspicuous dorsal projection	***T.bonifi* Lavesque, Hutchings, Daffe, Nygren & Londoño-Mesa, 2019**
15	Branchial ciliary tufts present	***T.gentili* Lavesque, Hutchings, Daffe, Nygren & Londoño-Mesa, 2019**
–	Branchial ciliary tufts absent	**16**
16	Most branchial lamellae with marginal papillae; mouth with upper lip elongated	***T.resomari* Lavesque, Hutchings, Daffe, Nygren & Londoño-Mesa, 2019**
–	Only anterior branchial lamellae with marginal papillae; upper lip not elongated	**17**
17	Thoracic uncini with capitium composed of 2–3 large teeth and subsequent ones much smaller	***T.ronningae* Parapar, Capa, Nygren & Moreira, 2020**
–	Thoracic uncini with capitium composed of 4 or 5 mid-sized teeth and following slightly smaller ones	**18**
18	Deep-water species; usually at depths below 200 m	***T.norvegica* Parapar, Capa, Nygren & Moreira, 2020**
–	Shallow-water species; mostly at depths above 100 m	***T.europaea* Lavesque, Hutchings, Daffe, Nygren & Londoño-Mesa, 2019 and *T.scotica* Parapar, Capa, Nygren & Moreira, 2020**

## ﻿Discussion

### ﻿Species groups

According to Nygren et al. (2018), *Terebellides* is divided in four main groups of species: A, B, C and D, which differ from each other by several morphological characters. Species of Group A were studied by Parapar et al. (2020a). Species in Group B are characterised by small-medium body length (5.0–35.0 mm), branchiae of type 2 or 3 with incompletely fused or free lobes (only fused at their base), long posterior filaments in ventral lobes, thoracic uncini of type 3 or 4 and abdominal uncini of type 2. *Terebellideslavesquei* sp. nov. belongs to this group and is also characterised by the lack of papillae on margins of branchial lamellae and by having branchiae of type 2 and thoracic uncini of type 3. Group C is defined by having thoracic uncini of type 3 only and abdominal uncini of type 2. Finally, Group D species are of medium length and bear white ventral colouration in anterior chaetigers, branchiae of type 2 with incompletely fused lobes, ventral branchial lobes with posterior filaments, ciliary tufts in the inner face of branchial lamellae, thoracic uncini of type 1 and abdominal uncini of type 1A.

### ﻿Integrative taxonomy

The apparently morphological homogeneity of members of *Terebellides* has hidden an unexpected species richness in the Northeast Atlantic. Species delimitation analyses of DNA sequence data have allowed to reveal some of the *Terebellides* species that otherwise would have gone unnoticed (Nygren et al. 2018; Lavesque et al. 2019; Parapar et al. 2020a). Moreover, molecular data have provided further evidence of species hypothesis diagnosed solely based on morphological features (e.g., Gagaev 2009; Parapar et al. 2011, 2016c; Parapar and Hutchings 2014).

An integrative approach, that aims at considering different sources of evidence, has become a common and grounded method for general species delineation (Dayrat 2005; Schlick-Steiner et al. 2010) including marine annelids (Capa et al. 2010, 2013; Capa and Murray 2015; Aguado et al. 2019; Kara et al. 2020; Teixeira et al. 2020). In addition, the integration of a variety of types of data has been used in formal species descriptions, a crucial step that includes providing a new name and facilitates communication about these entities (Goldstein and DeSalle 2011) and their diagnoses, which allows the correct identification for the species (Pante et al. 2015). In fact, the inclusion of DNA sequence information in formal species descriptions or diagnoses has been revealed as a useful practice to identify taxonomic groups (Renner 2016), especially with a high proportion of cryptic and pseudo-cryptic species such as it happens in annelids (Halt et al. 2009; Nygren and Pleijel 2011; Parapar et al. 2020a; Grosse et al. 2021).

In the present study, following the aims and methods of the similar previous study by Parapar et al. (2020a), who dealt with species belonging to Group A, several of the Northeast Atlantic Ocean *Terebellides* molecular lineages recovered within Groups B, C and D and compatible with a hypothetical species rank (after Nygren et al. 2018) are newly described, including morphological and COI sequence traits allowing to distinguish them from other congeners. In addition, a new species, *Terebellideslavesquei* sp. nov. is also described following the same approach. The difference between the present work and the previous (Parapar et al. 2020a) relies on the recognition of the diagnostic COI nucleotides for the species described. Parapar et al. (2020a) provided a list of unequivocal single nucleotides (autapomorphies) in specific positions of the alignment while in the present study a short sequence of nucleotides is provided to ease the identification along the alignment, and these include a unique combination of single nucleotides together with others that do not show variation within the sequences available.

Group B comprises eight species; one of them was identified herein as *T.atlantis*, matching the diagnostic characters and distribution of *T.atlantis*, originally described by Williams (1984) and from specimens collected in deep Icelandic waters by Parapar et al. (2011). A second species was recognised as *T.shetlandica* according to the description by Parapar et al. (2016a). The remaining six species represent undescribed taxa that will be dealt with elsewhere. Group C was composed by three species; one species was identified as *T.irinae* and the other two represent undescribed taxa that will be dealt with elsewhere. Finally, Group D comprises three species: *T.gracilis*, that matches the diagnostic characters and distribution originally described by Malm (1874), *T.williamsae* following the original description by Jirkov (1989), and one undescribed taxon that will be dealt with elsewhere.

### ﻿Species distributions

The range of distribution of all nominal species identified here is expanded. Nygren et al. (2018) pointed out that species such as *T.shetlandica* and *T.atlantis* have a wide distribution and were more frequent in samples, while *T.lavesquei* sp. nov. seems restricted to the Norwegian and Swedish coast. Some species were found at shallow depths, reaching the continental shelf border (0–200 m) such as *T.shetlandica*, while *T.atlantis* and *T.lavesquei* sp. nov. were found at depths below 200 m. *Terebellidesatlantis* showed the wider bathymetric distribution (219–2750 m deep) among the species of this group. *Terebellidesirinae* appeared at depths below 4000 m and its distribution seems to be restricted to the Arctic Ocean. Species of Group D, *T.williamsae* and *T.gracilis*, show a wide geographic and bathymetric distribution.

### ﻿Comparisons with other NEA species of the genus *Terebellides*

Lavesque et al. (2019) described eight species from the Atlantic and Mediterranean coasts of France (see Key above), six of them belong to Group A sensu Nygren et al. (2018) and two are morphologically similar to those of Groups B and D, namely *T.ceneresi* and *T.parapari*. Lavesque et al. (2019) and Parapar et al. (2020a) considered *T.parapari* as related to Group B and particularly to *T.shetlandica*. *Terebellidesparapari* shares with *T.shetlandica* and *T.atlantis* the presence of branchiae of type 3 and branchial lobes that are free from each other; *T.parapari* also shares with *T.shetlandica*, *T.lavesquei* sp. nov. and *T.atlantis* the presence of posterior filaments in lower branchial lobes, thoracic uncini of type 3 and abdominal uncini of type 2. However, branchial filaments in *T.shetlandica*, *T.lavesquei* sp. nov. and *T.atlantis* are longer and all notochaetae are of similar length while in *T.parapari* notochaetae in TC 1 are longer than subsequent ones.

On the other hand, *T.ceneresi* shares many morphological similarities with *T.williamsae* and *T.gracilis* and therefore was related to Group D by Lavesque et al. (2019) and Parapar et al. (2020a). These three species show white ventral colouration in anterior thoracic chaetigers, but in *T.williamsae* it is present in TC 1–4 while in *T.ceneresi* and *T.gracilis* is present only in TC 4. Other shared characters are the presence of ciliary tufts in the inner face of branchial lamellae and abdominal uncini of type 1A. However, *T.ceneresi* lacks the anterior branchial lobe (5^th^ lobe) that is present in *T.gracilis* and *T.williamsae*; the branchial lobes of *T.ceneresi* are not fused while in *T.williamsae* and *T.gracilis* they are partially fused. Finally, *T.ceneresi* bears thoracic uncini of type 3 whereas *T.williamsae* and *T.gracilis* bear type 1.

Among the remaining clades that will be described elsewhere, clades 4, 14 and 26 do not correspond either to *T.atlantis* or *T.shetlandica* because of differences in the branchiae type (i.e., type 2: incompletely fused lobes) and the absence of posterior filaments in branchial ventral lobes; they also differ in geographic distribution, being the aforementioned clades restricted to some areas in NEA. Likewise, clade 25 does not fit within *T.irinae* due to being medium sized and by having branchiae of type 1 (=large lobes almost totally fused). Finally, clade 15 does not match either to *T.williamsae* or *T.gracilis* because of having a ventral colouration extending across more segments (TC 1–10 vs TC 1–4 or TC 4 in *T.gracilis* and *T.williamsae* respectively).

### ﻿Characters and identification key

In this work, the following characters have been studied in all specimens: morphology of branchiae (sensu Parapar et al. 2016a), types of thoracic uncini (sensu Parapar et al. 2020b), abdominal uncini (sensu Parapar et al. 2020a), MG staining patterns (sensu Schüller and Hutchings 2010, 2013), and geographic and bathymetric distributions.

Among the species studied here, branchiae of *T.lavesquei* sp. nov., *T.williamsae*, and *T.gracilis* correspond to type 2, *T.shetlandica* and *T.atlantis* to type 3, and *T.irinae* to type 4. Regarding thoracic uncini, *T.williamsae* and *T.gracilis* have type 1, *T.lavesquei* sp. nov., *T.atlantis*, and *T.irinae* have type 3 and *T.shetlandica* bears type 4. Finally, considering abdominal uncini, *T.williamsae* and *T.gracilis* have type 1A and *T.shetlandica*, *T.lavesquei* sp. nov., *T.atlantis*, and *T.irinae* have type 2.

Schüller and Hutchings (2010, 2013) defined several types of MG staining patterns according to the presence or absence of coloured bands in the segments along the body and their solid/striped appearance. The patterns observed in the species studied here are similar to those patterns described by Schüller and Hutchings (2010, 2013): *T.shetlandica* and *T.irinae* agree to pattern 1, *T.williamsae* and *T.gracilis* to pattern 2, and *T.lavesquei* sp. nov. and *T.atlantis* to pattern 9. However, we found that species with types 2 and 9 bear a J-shaped glandular region that is composed of three segments instead of only one as reported by Schüller and Hutchings (2010, 2013). These variations suggest that new staining patterns with taxonomic relevance might be determined when specimens from elsewhere are studied.

The species key is an update to those by Lavesque et al. (2019) and Parapar et al. (2020b) but still does not allow for morphological discrimination between three species (*T.norvegica*, *T.europaea*, and *T.scotica*). At present, these species can be differentiated only genetically and according to their geographical or bathymetric distributions.

## ﻿Conclusions

A total of five nominal species has been identified as belonging to *Terebellides* Groups B, C, and D (according to Nygren et al. 2018): *Terebellidesgracilis* Malm, 1874, *Terebellidesatlantis* Williams, 1984, *Terebellideswilliamsae* Jirkov, 1989, *Terebellidesirinae* Gagaev, 2009 and *Terebellidesshetlandica* Parapar, Moreira & O’Reilly, 2016, and a new species is here described as *Terebellideslavesquei* sp. nov. Other species outlined by species delimitation analyses within these Groups will be either described elsewhere or would require additional material to be found.

The five species identified herein have been characterised based on morphological and molecular characters. The most relevant morphological features discriminating between species are branchial shape, ventral pigmentation of anterior thoracic chaetigers in live and fixed specimens, and the morphology of thoracic and abdominal uncini. For the molecular recognition of the species described, short sequences of nucleotides among the COI alignment have been provided as diagnostic to ease the identification.

## Supplementary Material

XML Treatment for Terebellides

XML Treatment for Terebellides

XML Treatment for Terebellides shetlandica

XML Treatment for Terebellides lavesquei

XML Treatment for Terebellides atlantis

XML Treatment for Terebellides

XML Treatment for Terebellides irinae

XML Treatment for Terebellides

XML Treatment for Terebellides williamsae

XML Treatment for Terebellides gracilis

## Supplementary materials

Supplementary material 1 Abiotic data and more information of the material used Data type Abiotic data Explanation note The table shows the abiotic data and more information of the material used: Specimen voucher, Site, Geographic area, Locality, Latitude, Longitude, Depth, Collecting date, Habitat, Remarks and figures. https://binary.pensoft.net/file/775035This dataset is made available under the Open Database License (http://opendatacommons.org/licenses/odbl/1.0/). The Open Database License (ODbL) is a license agreement intended to allow users to freely share, modify, and use this Dataset while maintaining this same freedom for others, provided that the original source and author(s) are credited.María Barroso, Juan Moreira, María Capa, Arne Nygren, Julio Parapar

Supplementary material 2 List of COI sequences considered in present study Data type Molecular data Explanation note List of COI sequences considered in present study (Group B, C and D), museum vouchers and GenBank accession numbers. https://binary.pensoft.net/file/775036This dataset is made available under the Open Database License (http://opendatacommons.org/licenses/odbl/1.0/). The Open Database License (ODbL) is a license agreement intended to allow users to freely share, modify, and use this Dataset while maintaining this same freedom for others, provided that the original source and author(s) are credited.María Barroso, Juan Moreira, María Capa, Arne Nygren, Julio Parapar
