# Supplementary material for: ﻿A further step towards the characterisation of Terebellides (Annelida, Trichobranchidae) diversity in the Northeast Atlantic, with the description of a new species
Source: Zookeys. 2022 Nov 28;1132:85–126. doi: 10.3897/zookeys.1132.91244 (PMC9836732; doi:10.3897/zookeys.1132.91244)
Supplement: Supplementary material 1 — Abiotic data and more information of the material used [file zookeys-1132-085_article-91244__-s001.docx]

Supplementary Material – Table S1

n.d. **–** no data

| **Specimen voucher** | **Site** | **Geographic area** | **Locality** | **Latitude** | **Longitude** | **Depth (m)** | **Collecting date** | **Habitat** | **Remarks** | **Figures** |
| --- | --- | --- | --- | --- | --- | --- | --- | --- | --- | --- |
| **clade 1** |  |  |  |  |  |  |  |  |  |  |
| ZMBN116171 | n.d. | Koster Area | SW Yttre Vattenholmen | 58.87417 | 11.09472 | 71 - 62 | 08/04/2008 | Mud, soft bottom |  | Fig. 2A |
| ZMBN116172 | n.d. | Koster Area | SW Yttre Vattenholmen | 58.87417 | 11.09472 | 71 - 62 | 08/04/2008 | Mud, soft bottom |  |  |
| ZMBN116173 | n.d. | Koster Area | SW Yttre Vattenholmen | 58.87417 | 11.09472 | 71 - 62 | 08/04/2008 | Mud, soft bottom |  |  |
| ZMBN116174 | n.d. | Koster Area | SW Yttre Vattenholmen | 58.87417 | 11.09472 | 71 - 62 | 08/04/2008 | Mud, soft bottom |  |  |
| ZMBN116175 | n.d. | Koster Area | SW Yttre Vattenholmen | 58.87417 | 11.09472 | 71 - 62 | 08/04/2008 | Mud, soft bottom |  |  |
| ZMBN116176 | n.d. | Koster Area | SW Yttre Vattenholmen | 58.87417 | 11.09472 | 71 - 62 | 08/04/2008 | Mud, soft bottom |  |  |
| ZMBN116177 | n.d. | Koster Area | SW Yttre Vattenholmen | 58.87417 | 11.09472 | 71 - 62 | 08/04/2008 | Mud, soft bottom |  |  |
| ZMBN116178 | n.d. | Koster Area | SW Yttre Vattenholmen | 58.87417 | 11.09472 | 71 - 62 | 08/04/2008 | Mud, soft bottom |  |  |
| ZMBN116179 | n.d. | Koster Area | SW Yttre Vattenholmen | 58.87417 | 11.09472 | 71 - 62 | 08/04/2008 | Mud, soft bottom |  |  |
| ZMBN116180 | n.d. | Koster Area | SW Yttre Vattenholmen | 58.87417 | 11.09472 | 71 - 62 | 08/04/2008 | Mud, soft bottom |  |  |
| ZMBN116181 | n.d. | Koster Area | SW Yttre Vattenholmen | 58.87417 | 11.09472 | 71 - 62 | 08/04/2008 | Mud, soft bottom |  | Figs 2B, 5B, C, F |
| GNM15106 | SK146 | Skagerrak | n.d. | 58.14456 | 10.71923 | 245 - 297 | 12/06/2008 | SB (three buckets of mud) |  |  |
| ZMBN116182 | n.d. | North Sea | n.d. | 57.98075 | -2.83515 | 76 | 07/2008 | Sand, fine gravel |  |  |
| ZMBN116183 | n.d. | North Sea | n.d. | 57.98075 | -2.83515 | 76 | 07/2008 | Sand, fine gravel |  |  |
| ZMBN116184 | n.d. | North Sea | n.d. | 57.98075 | -2.83515 | 76 | 07/2008 | Sand, fine gravel |  |  |
| ZMBN116185 | n.d. | Koster Area | n.d. | 58.86667 | 11.10000 | 60 - 80 | 04/2005 | Mud, soft bottom |  |  |
| ZMBN116186 | n.d. | Koster Area | n.d. | 58.86667 | 11.10000 | 60 - 80 | 04/2005 | Mud, soft bottom |  | Figs 3A, 4A |
| ZMBN116187 | n.d. | Koster Area | n.d. | 58.86667 | 11.10000 | 60 - 80 | 04/2005 | Mud, soft bottom |  |  |
| ZMBN116188 | n.d. | Koster Area | n.d. | 58.86667 | 11.10000 | 60 - 80 | 04/2005 | Mud, soft bottom |  |  |
| ZMBN116189 | n.d. | Koster Area | n.d. | 58.86667 | 11.10000 | 60 - 80 | 04/2005 | Mud, soft bottom |  |  |
| ZMBN116190 | n.d. | Koster Area | n.d. | 58.86667 | 11.10000 | 60 - 80 | 04/2005 | Mud, soft bottom |  |  |
| ZMBN116191 | n.d. | Koster Area | n.d. | 58.86667 | 11.10000 | 60 - 80 | 04/2005 | Mud, soft bottom |  |  |
| ZMBN116192 | n.d. | Koster Area | n.d. | 58.86667 | 11.10000 | 60 - 80 | 04/2005 | Mud, soft bottom |  |  |
| ZMBN116193 | n.d. | Koster Area | n.d. | 58.86667 | 11.10000 | 60 - 80 | 04/2005 | Mud, soft bottom |  |  |
| ZMBN116194 | n.d. | Koster Area | n.d. | 58.86667 | 11.10000 | 60 - 80 | 04/2005 | Mud, soft bottom |  |  |
| ZMBN116195 | n.d. | Koster Area | n.d. | 58.86667 | 11.10000 | 60 - 80 | 04/2005 | Mud, soft bottom |  |  |
| ZMBN116196 | n.d. | Koster Area | n.d. | 58.86667 | 11.10000 | 60 - 80 | 04/2005 | Mud, soft bottom |  |  |
| ZMBN116197 | n.d. | Koster Area | n.d. | 58.86667 | 11.10000 | 60 - 80 | 04/2005 | Mud, soft bottom |  |  |
| ZMBN116198 | n.d. | Koster Area | n.d. | 58.86667 | 11.10000 | 60 - 80 | 04/2005 | Mud, soft bottom |  |  |
| ZMBN116199 | n.d. | Koster Area | n.d. | 58.86667 | 11.10000 | 60 - 80 | 04/2005 | Mud, soft bottom |  |  |
| ZMBN116200 | n.d. | Koster Area | n.d. | 58.86667 | 11.10000 | 60 - 80 | 04/2005 | Mud, soft bottom |  |  |
| ZMBN116201 | n.d. | Koster Area | n.d. | 58.86667 | 11.10000 | 60 - 80 | 04/2005 | Mud, soft bottom |  |  |
| ZMBN116202 | n.d. | Koster Area | n.d. | 58.86667 | 11.10000 | 60 - 80 | 04/2005 | Mud, soft bottom |  |  |
| ZMBN116203 | n.d. | Koster Area | n.d. | 58.86667 | 11.10000 | 60 - 80 | 04/2005 | Mud, soft bottom |  |  |
| ZMBN116204 | n.d. | Koster Area | n.d. | 58.86667 | 11.10000 | 60 - 80 | 04/2005 | Mud, soft bottom |  | Fig. 5A |
| ZMBN116205 | n.d. | Koster Area | n.d. | 58.86667 | 11.10000 | 60 - 80 | 04/2005 | Mud, soft bottom |  |  |
| ZMBN116206 | n.d. | Koster Area | n.d. | 58.86667 | 11.10000 | 60 - 80 | 04/2005 | Mud, soft bottom |  |  |
| ZMBN116207 | n.d. | Arendal | Ærøydypet | 58.40663 | 8.77758 | 90 - 100 | 26/05/2011 | Mud, soft bottom |  |  |
| ZMBN116208 | n.d. | Arendal | Ærøydypet | 58.40663 | 8.77758 | 90 - 100 | 26/05/2011 | Mud, soft bottom |  |  |
| ZMBN116209 | n.d. | Arendal | Ærøydypet | 58.40663 | 8.77758 | 90 - 100 | 26/05/2011 | Mud, soft bottom |  |  |
| ZMBN116210 | n.d. | Arendal | Ærøydypet | 58.40663 | 8.77758 | 90 - 100 | 26/05/2011 | Mud, soft bottom |  |  |
| ZMBN116211 | n.d. | Grimstad | Ryvingdypet | 58.36978 | 8.72617 | 190 | 28/05/2011 | Mud with dead algae |  |  |
| ZMBN116212 | n.d. | Grimstad | Ryvingdypet | 58.36978 | 8.72617 | 190 | 28/05/2011 | Mud with dead algae |  |  |
| ZMBN116213 | n.d. | Grimstad | Ryvingdypet | 58.36978 | 8.72617 | 190 | 28/05/2011 | Mud with dead algae |  |  |
| ZMBN116214 | n.d. | Sandefjord | Håholmboen, Sandefjordsfj | 59.05485 | 10.25047 | 63 - 75 | 29/05/2011 | Mud, soft bottom |  |  |
| ZMBN116215 | n.d. | Sandefjord | Håholmboen, Sandefjordsfj | 59.05485 | 10.25047 | 63 - 75 | 29/05/2011 | Mud, soft bottom |  |  |
| ZMBN116216 | n.d. | Sandefjord | Håholmboen, Sandefjordsfj | 59.05485 | 10.25047 | 63 - 75 | 29/05/2011 | Mud, soft bottom |  |  |
| ZMBN116217 | n.d. | Sandefjord | Håholmboen, Sandefjordsfj | 59.05485 | 10.25047 | 63 - 75 | 29/05/2011 | Mud, soft bottom |  |  |
| ZMBN116218 | n.d. | Sandefjord | Håholmboen, Sandefjordsfj | 59.05485 | 10.25047 | 63 - 75 | 29/05/2011 | Mud, soft bottom |  |  |
| ZMBN116219 | n.d. | Sandefjord | Håholmboen, Sandefjordsfj | 59.05485 | 10.25047 | 63 - 75 | 29/05/2011 | Mud, soft bottom |  | Fig. 5D, E |
| ZMBN116220 | n.d. | Sandefjord | Håholmboen, Sandefjordsfj | 59.05485 | 10.25047 | 63 - 75 | 29/05/2011 | Mud, soft bottom |  |  |
| GNM14640 | SK46 | Skagerrak | n.d. | 58.0081 | 11.20107 | 85 - 98 | 23/08/2006 | Sand, mud, gravel, stones |  |  |
| GNM14642 | KA31 | Kattegatt | Between Anholt and Tylösand | 56.68285 | 12.10700 | 30 - 33 | 23/05/2007 | Soft bottom |  |  |
| GNM14643 | KA31 | Kattegatt | Between Anholt and Tylösand | 56.68452 | 12.10962 | 29 - 32 | 23/05/2007 | Soft bottom, clay with sand |  |  |
| GNM14643:1 | KA31 | Kattegatt | Between Anholt and Tylösand | 56.68452 | 12.10962 | 29 - 32 | 23/05/2007 | Soft bottom, clay with sand |  |  |
| GNM14649 | SK177 | Skagerrak | W Grebbestad | 58.68122 | 11.11432 | 53 - 54 | 16/06/2008 | Soft bottom, mixed bottom |  |  |
| ZMBN116221 | HM2014/06-32 | Rogaland | Karmøysundet | 59.28789 | 5.32506 | 74 - 79 | 08/06/2014 | Mud |  |  |
| ZMBN116222 | HM2014/06-32 | Rogaland | Karmøysundet | 59.28790 | 5.32507 | 74 - 79 | 08/06/2014 | Mud |  |  |
| ZMBN116223 | HM2014/06-32 | Rogaland | Karmøysundet | 59.28791 | 5.32508 | 74 - 79 | 08/06/2014 | Mud |  |  |
| ZMBN116224 | HM2014/06-46 | Rogaland | Kvitsøy, S | 59.02985 | 5.44881 | 58 - 60 | 10/06/2014 | Stein, shell, grus, sand |  |  |
| ZMBN116225 | HM2014/06-46 | Rogaland | Kvitsøy, S | 59.02986 | 5.44882 | 58 - 60 | 10/06/2014 | Stein, shell, grus, sand |  |  |
| ZMBN116226 | HM2014/06-44 | Rogaland | Kvitsøy, S | 59.02712 | 5.45419 | 64 | 10/06/2014 | Sand with mud |  |  |
| ZMBN116227 | HM2014/06-46 | Rogaland | Kvitsøy, S | 59.02985 | 5.44881 | 58 - 60 | 10/06/2014 | Stein, shell, grus, sand |  |  |
| ZMBN116228 | HM2014/06-46 | Rogaland | Kvitsøy, S | 59.02985 | 5.44881 | 58 - 60 | 10/06/2014 | Stein, shell, grus, sand |  |  |
| NTNU-VM61384 | n.d. | Sør-Trøndelag | Trondheimsfjorden | 63.445 | 10.17100 | 40 | 17/01/2013 | n.d. |  |  |
| NTNU-VM61385 | n.d. | Sør-Trøndelag | Trondheimsfjorden | 63.445 | 10.17100 | 40 | 17/01/2013 | n.d. |  |  |
| NTNU-VM59990 | St. Ra1 | North Sea | Region 3 | 56.75000 | 3.00000 | 111 | 07/02/2008 | Soft bottom |  |  |
| ZMBN116229 | HB2008.03.17-2 | Hordaland | Bergen | 60.26915 | 5.11583 | 102 | 17/03/2008 | n.d. |  |  |
| ZMBN116230 | HB2008.03.17-2 | Hordaland | Bergen | 60.26915 | 5.11583 | 102 | 17/03/2008 | n.d. |  |  |
| ZMBN116231 | HB2008.03.17-2 | Hordaland | Bergen | 60.26915 | 5.11583 | 102 | 17/03/2008 | n.d. |  |  |
| ZMBN116232 | VTD-11_14 | n.d. | Northern North Sea | 61.34553 | 2.06935 | 246 | 31/05/2014 | n.d. |  |  |
| ZMBN116233 | VTD-11_14 | n.d. | Northern North Sea | 61.34553 | 2.06935 | 246 | 31/05/2014 | n.d. |  |  |
| ZMBN116234 | VTD-11_14 | n.d. | Northern North Sea | 61.34553 | 2.06935 | 246 | 31/05/2014 | n.d. |  |  |
| ZMBN116235 | HB2006.05.02-4 | Hordaland | Bergen | 60.2691 | 5.11570 | 98 | 02/05/2006 | n.d. |  |  |
| ZMBN116236 | HB2006.05.02-4 | Hordaland | Bergen | 60.2691 | 5.11570 | 98 | 02/05/2006 | n.d. |  |  |
| ZMBN116237 | HB2006.05.02-4 | Hordaland | Bergen | 60.2691 | 5.11570 | 98 | 02/05/2006 | n.d. |  |  |
| ZMBN116238 | HB2007.06.28-2 | Hordaland | Bergen | 60.21465 | 5.34720 | 25 - 47 | 28/06/2007 | n.d. |  |  |
| ZMBN116239 | HB2007.06.28-2 | Hordaland | Bergen | 60.21465 | 5.34720 | 25 - 47 | 28/06/2007 | n.d. |  |  |
| ZMBN116240 | HB2007.06.28-2 | Hordaland | Bergen | 60.21465 | 5.34720 | 25 - 47 | 28/06/2007 | n.d. |  |  |
| ZMBN116241 | HB 2014-05-19, Polychaeta 2, 19/5-14 | Hordaland | Bergen | 60.23330 | 5.28042 | 103 | 19/05/2014 | Clay |  |  |
| ZMBN116242 | HM2012/07-01 | Sogn-Møre | n.d. | 61.80178 | 5.08135 | 370 - 375 | 20/07/2012 | n.d. |  |  |
| ZMBN116243 | HM2012/07-01 | Sogn-Møre | n.d. | 61.80178 | 5.08135 | 370 - 375 | 20/07/2012 | n.d. |  |  |
| **Clade 2** |  |  |  |  |  |  |  |  |  |  |
| **Specimen voucher** | **Site** | **Geographic area** | **Locality** | **Latitude** | **Longitude** | **Depth (m)** | **Collecting date** | **Habitat** | **Remarks** | **Figures** |
| GNM15107 | SK146 | Skagerrak | n.d. | 58.14457 | 10.71923 | 245 - 297 | 12/06/2008 | Mud |  |  |
| GNM15108 | SK164 | Skagerrak | n.d. | 58.45702 | 10.54635 | 224 - 286 | 14/06/2008 | Hard bottom, with some mud |  | Fig. 2D |
| GNM15109 | SK148 | Skagerrak | n.d. | 58.19173 | 10.66480 | 237 - 277 | 12/06/2008 | Mud, silt |  | Fig. 2E |
| GNM14625:1 | FL27 | Kattegatt | Fladen | 57.19717 | 11.82517 | 38 | 17/06/2005 | Silt with sand |  |  |
| ZMBN116247 | R1331-BT501 | Finnmark | n.d. | 71.32100 | 29.19650 | 362 | 24/04/2014 | Mud, soft bottom |  |  |
| ZMBN116248 | R1331-BT 501 | Finnmark | n.d. | 71.32100 | 29.19650 | 362 | 24/04/2014 | Mud, soft bottom |  |  |
| GNM14639 | SK43 | Skagerrak | n.d. | 58.43017 | 10.57998 | 335 - 248 | 22/08/2006 | Soft clay |  |  |
| ZMBN116249 | R1213-469 | Finnmark Ost | n.d. | 70.77383 | 30.78117 | 377 - 378 | 17/08/2013 | n.d. |  | Figs 14A-C, 15 |
| ZMBN116250 | R1158-83 | Finnmark | n.d. | 71.90850 | 33.44717 | 219 - 220 | 06/08/2013 | n.d. |  |  |
| ZMBN116251 | R1129-70 | n.d. | Skjoldryggen | 65.28217 | 6.28326 | 357 - 369 | 24/06/2013 | n.d. |  | Fig. 14D, E |
| ZMBN116252 | R1137-77 | Finnmark | n.d. | 72.57905 | 32.38725 | 271 - 272 | 03/08/2013 | n.d. |  |  |
| ZMBN116253 | R1137-77 | Finnmark | n.d. | 72.57905 | 32.38725 | 271 - 272 | 03/08/2013 | n.d. |  |  |
| ZMBN116254 | R1137-77 | Finnmark | n.d. | 72.57905 | 32.38725 | 271 - 272 | 03/08/2013 | n.d. |  |  |
| ZMBN116255 | R1230-95 | Finnmark OstOst | n.d. | 70.11767 | 31.35034 | 303 - 304 | 19/08/2013 | n.d. |  |  |
| ZMBN116256 | R1180-86 | Finnmark | n.d. | 71.61527 | 32.99710 | 305 - 306 | 09/08/2013 | n.d. |  |  |
| ZMBN116257 | R1137-456 | Finnmark | n.d. | 72.58050 | 32.38367 | 271 - 272 | 03/08/2013 | n.d. |  |  |
| ZMBN116258 | R1137-456 | Finnmark | n.d. | 72.58050 | 32.38367 | 271 - 272 | 03/08/2013 | n.d. |  |  |
| ZMBN116259 | R1137-456 | Finnmark | n.d. | 72.58050 | 32.38367 | 271 - 272 | 03/08/2013 | n.d. |  |  |
| ZMBN116260 | R1137-456 | Finnmark | n.d. | 72.58050 | 32.38367 | 271 - 272 | 03/08/2013 | n.d. |  |  |
| ZMBN116261 | R1137-456 | Finnmark | n.d. | 72.58050 | 32.38367 | 271 - 272 | 03/08/2013 | n.d. |  |  |
| ZMBN116262 | R1137-456 | Finnmark | n.d. | 72.58050 | 32.38367 | 271 - 272 | 03/08/2013 | n.d. |  |  |
| ZMBN116263 | R1137-456 | Finnmark | n.d. | 72.58050 | 32.38367 | 271 - 272 | 03/08/2013 | n.d. |  |  |
| ZMBN116264 | R1137-456 | Finnmark | n.d. | 72.58050 | 32.38367 | 271 - 272 | 03/08/2013 | n.d. |  |  |
| ZMBN116265 | R1137-456 | Finnmark | n.d. | 72.58050 | 32.38367 | 271 - 272 | 03/08/2013 | n.d. |  |  |
| ZMBN116266 | R1174-85 | Finnmark | n.d. | 71.61817 | 32.23133 | 297 - 298 | 08/08/2013 | n.d. |  |  |
| ZMBN116267 | R1174-85 | Finnmark | n.d. | 71.61817 | 32.23133 | 297 - 298 | 08/08/2013 | n.d. |  |  |
| ZMBN116268 | R1093-59 | n.d. | Skjoldryggen | 65.94317 | 5.83320 | 610 - 612 | 17/06/2013 | n.d. |  |  |
| ZMBN116269 | R1093-59 | n.d. | Skjoldryggen | 65.94317 | 5.83320 | 610 - 612 | 17/06/2013 | n.d. |  | Fig. 3E |
| ZMBN116270 | R1180-463 | Finnmark | n.d. | 71.61416 | 33.00411 | 305 | 09/08/2013 | n.d. |  |  |
| ZMBN116271 | R1180-463 | Finnmark | n.d. | 71.61416 | 33.00411 | 305 | 09/08/2013 | n.d. |  |  |
| ZMBN116272 | M09JAN0193-8 | Finnmark | Porsangerfjorden | 70.35324 | 25.26368 | 178 | 30/05/2009 | n.d. |  |  |
| **Clade 3** |  |  |  |  |  |  |  |  |  |  |
| **Specimen voucher** | **Site** | **Geographic area** | **Locality** | **Latitude** | **Longitude** | **Depth (m)** | **Collecting date** | **Habitat** | **Remarks** | **Figures** |
| GNM15110 | st 12 | Skagerrak | n.d. | 58.3532 | 10.3300 | 390 - 406 | 13/05/2009 | Fine mud |  | Fig.16A-C |
| GNM15111 | SK148 | Skagerrak | n.d. | 58.19173 | 10.6648 | 237 - 277 | 12/06/2008 | Mud, silt |  | Fig. 2F |
| ZMBN116273 | R1093-59 |  | Skjoldryggen | 65.94317 | 5.83320 | 610 - 612 | 17/06/2013 | n.d. |  |  |
| NTNU-VM68196 | st 2013003 | Sor-Trondelag | Trondheimsfjorden | 63.4770 | 9.92900 | 534 | 17/01/2013 | n.d. |  |  |
| ZMBN116274 | HM2012-11, 06RP, Sognefjorden | Sogn | Sognefjorden2-Havnaneset | 61.14484 | 5.91575 | 1259 - 1268 | 16/11/2012 | n.d. |  |  |
| ZMBN116275 | HM2012-11, 06RP, Sognefjorden | Sogn | Sognefjorden2-Havnaneset | 61.14484 | 5.91575 | 1259 - 1268 | 16/11/2012 | n.d. |  |  |
| ZMBN116276 | HM2012/07-04 | Sogn-Møre | n.d. | 61.82371 | 5.21031 | 446 - 453 | 20/07/2012 | n.d. |  |  |
| ZMBN116277 | HM2012/11-03 | Sogn | slope-south of Nesholmen | 61.08952 | 5.21063 | 300 - 619 | 15/11/2012 | n.d. |  |  |
| ZMBN116278 | HM2012/11-23 | Sogn | Lustrafjorden-Nattropefjorden | 61.43212 | 7.47763 | 327 - 337 | 18/11/2012 | n.d. |  |  |
| ZMBN116279 | HM2012/11-23 | Sogn | Lustrafjorden-Nattropefjorden | 61.43212 | 7.47763 | 327 - 337 | 18/11/2012 | n.d. |  |  |
| ZMBN116280 | HM2012/11-23 | Sogn | Lustrafjorden-Nattropefjorden | 61.43212 | 7.47763 | 327 - 337 | 18/11/2012 | n.d. |  |  |
| ZMBN116281 | HM2012/11-23 | Sogn | Lustrafjorden-Nattropefjorden | 61.43212 | 7.47763 | 327 - 337 | 18/11/2012 | n.d. |  |  |
| ZMBN116282 | HM2012/11-23 | Sogn | Lustrafjorden-Nattropefjorden | 61.43212 | 7.47763 | 327 - 337 | 18/11/2012 | n.d. |  | Figs 16A-C, 17, 18D-F |
| ZMBN116283 | HM2012/11-23 | Sogn | Lustrafjorden-Nattropefjorden | 61.43212 | 7.47763 | 327 - 337 | 18/11/2012 | n.d. |  | Figs 3F, 4D |
| ZMBN116284 | HM2012/11-23 | Sogn | Lustrafjorden-Nattropefjorden | 61.43212 | 7.47763 | 327 - 337 | 18/11/2012 | n.d. |  |  |
| ZMBN116285 | HM2012/11-23 | Sogn | Lustrafjorden-Nattropefjorden | 61.43212 | 7.47763 | 327 - 337 | 18/11/2012 | n.d. |  |  |
| ZMBN116286 | HM2012/11-23 | Sogn | Lustrafjorden-Nattropefjorden | 61.43212 | 7.47763 | 327 - 337 | 18/11/2012 | n.d. |  |  |
| ZMBN116287 | HM2012/11-23 | Sogn | Lustrafjorden-Nattropefjorden | 61.43212 | 7.47763 | 327 - 337 | 18/11/2012 | n.d. |  |  |
| ZMBN116288 | HM2012/11-23 | Sogn | Lustrafjorden-Nattropefjorden | 61.43212 | 7.47763 | 327 - 337 | 18/11/2012 | n.d. |  |  |
| ZMBN116289 | HM2012/11-23 | Sogn | Lustrafjorden-Nattropefjorden | 61.43212 | 7.47763 | 327 - 337 | 18/11/2012 | n.d. |  |  |
| ZMBN116290 | HM2012/11-23 | Sogn | Lustrafjorden-Nattropefjorden | 61.43212 | 7.47763 | 327 - 337 | 18/11/2012 | n.d. |  |  |
| ZMBN116291 | HM2012/11-23 | Sogn | Lustrafjorden-Nattropefjorden | 61.43212 | 7.47763 | 327 - 337 | 18/11/2012 | n.d. |  |  |
| ZMBN116292 | HM2012/11-23 | Sogn | Lustrafjorden-Nattropefjorden | 61.43212 | 7.47763 | 327 - 337 | 18/11/2012 | n.d. |  |  |
| ZMBN116293 | HM2012/11-23 | Sogn | Lustrafjorden-Nattropefjorden | 61.43212 | 7.47763 | 327 - 337 | 18/11/2012 | n.d. |  |  |
| ZMBN116294 | HM2012/11-23 | Sogn | Lustrafjorden-Nattropefjorden | 61.43212 | 7.47763 | 327 - 337 | 18/11/2012 | n.d. |  |  |
| ZMBN116295 | HM2012/11-23 | Sogn | Lustrafjorden-Nattropefjorden | 61.43212 | 7.47763 | 327 - 337 | 18/11/2012 | n.d. |  |  |
| ZMBN116296 | HM2012/11-23 | Sogn | Lustrafjorden-Nattropefjorden | 61.43212 | 7.47763 | 327 - 337 | 18/11/2012 | n.d. |  |  |
| ZMBN116297 | HM2012/07-21 | Sogn-Møre | n.d. | 61.13339 | 5.16632 | 631 - 644 | 22/07/2012 | n.d. |  |  |
| ZMBN116298 | HM2012/07-21 | Sogn-Møre | n.d. | 61.13339 | 5.16632 | 631 - 644 | 22/07/2012 | n.d. |  |  |
| ZMBN116299 | HM2012/07-01 | Sogn-Møre | n.d. | 61.80178 | 5.08135 | 370 - 375 | 20/07/2012 | n.d. |  |  |
| ZMBN116300 | HM2012/07-01 | Sogn-Møre | n.d. | 61.80178 | 5.08135 | 370 - 375 | 20/07/2012 | n.d. |  |  |
| ZMBN116301 | HM2012/07-01 | Sogn-Møre | n.d. | 61.80178 | 5.08135 | 370 - 375 | 20/07/2012 | n.d. |  |  |
| ZMBN116302 | HM2012/07-01 | Sogn-Møre | n.d. | 61.80178 | 5.08135 | 370 - 375 | 20/07/2012 | n.d. |  |  |
| ZMBN116303 | HM2012/07-21 | Sogn-Møre | n.d. | 61.13339 | 5.16632 | 631 - 644 | 22/07/2012 | n.d. |  |  |
| ZMBN116304 | HM2012/07-21 | Sogn-Møre | n.d. | 61.13339 | 5.16632 | 631 - 644 | 22/07/2012 | n.d. |  |  |
| ZMBN116305 | HM2012/07-01 | Sogn-Møre | n.d. | 61.80178 | 5.08135 | 370 - 375 | 20/07/2012 | n.d. |  |  |
| ZMBN116306 | HM2012/07-01 | Sogn-Møre | n.d. | 61.80178 | 5.08135 | 370 - 375 | 20/07/2012 | n.d. |  |  |
| ZMBN116307 | HM2012/07-01 | Sogn-Møre | n.d. | 61.80178 | 5.08135 | 370 - 375 | 20/07/2012 | n.d. |  |  |
| ZMBN116308 | HB2007.06.26-06 | Hordaland | Langenuen | 59.99 | 5.35 | 250 | 26/06/2007 | n.d. |  |  |
| ZMBN116309 | HB2007.06.26-06 | Hordaland | Langenuen | 59.99 | 5.35 | 250 | 26/06/2007 | n.d. |  |  |
| ZMBN116310 | HB2007.06.26-06 | Hordaland | Langenuen | 59.99 | 5.35 | 250 | 26/06/2007 | n.d. |  |  |
| ZMBN116311 | HM2012-11, 06RP, Sognefjorden | Sogn | Sognefjorden2-Havnaneset | 61.14484 | 5.91575 | 1259 - 1268 | 16/11/2012 | n.d. |  |  |
| ZMBN116312 | HM2012-11, 06RP, Sognefjorden | Sogn | Sognefjorden2-Havnaneset | 61.14484 | 5.91575 | 1259 - 1268 | 16/11/2012 | n.d. |  |  |
| ZMBN116313 | HM2012-11, 06RP, Sognefjorden | Sogn | Sognefjorden2-Havnaneset | 61.14484 | 5.91575 | 1259 - 1268 | 16/11/2012 | n.d. |  | Fig. 18A-C |
| ZMBN116314 | HM2012-11, 06RP, Sognefjorden | Sogn | Sognefjorden2-Havnaneset | 61.14484 | 5.91575 | 1259 - 1268 | 16/11/2012 | n.d. |  |  |
| ZMBN116315 | HM2012-11, 06RP, Sognefjorden | Sogn | Sognefjorden2-Havnaneset | 61.14484 | 5.91575 | 1259 - 1268 | 16/11/2012 | n.d. |  |  |
| ZMBN116316 | HM2012-11, 06RP, Sognefjorden | Sogn | Sognefjorden2-Havnaneset | 61.14484 | 5.91575 | 1259 - 1268 | 16/11/2012 | n.d. |  |  |
| ZMBN116317 | HM2012-11, 06RP, Sognefjorden | Sogn | Sognefjorden2-Havnaneset | 61.14484 | 5.91575 | 1259 - 1268 | 16/11/2012 | n.d. |  |  |
| **Clade 5** |  |  |  |  |  |  |  |  |  |  |
| **Specimen voucher** | **Site** | **Geographic area** | **Locality** | **Latitude** | **Longitude** | **Depth (m)** | **Collecting date** | **Habitat** | **Remarks** | **Figures** |
| GNM15112 | SK146 | Skagerrak | n.d. | 58.14457 | 10.71923 | 245 - 297 | 12/06/2008 | Mud |  | Fig. 2C |
| GNM15113 | SK146 | Skagerrak | n.d. | 58.14457 | 10.71923 | 245 - 297 | 12/06/2008 | Mud |  |  |
| NTNU-VM68252 | st 2013004 | Sor-Trondelag | Trondheimsfjorden | 63.47900 | 10.21300 | 505 | 17/01/2013 | n.d. |  |  |
| NTNU-VM61386 | n.d. | Sor-Trondelag | Trondheimsfjorden | 63.47700 | 9.99290 | 534 | 17/01/2013 | n.d. |  |  |
| NTNU-VM61387 | n.d. | Sor-Trondelag | Trondheimsfjorden | 63.47900 | 10.21300 | 505 | 17/01/2013 | n.d. |  | Fig. 7A, C, D |
| ZMBN116319 | HB2005.04.15.3 | Hordaland | Bergen | 60.12000 | 5.07000 | 119 | 15/04/2005 | n.d. |  |  |
| ZMBN116320 | HB2005.04.15.3 | Hordaland | Bergen | 60.12000 | 5.07000 | 119 | 15/04/2005 | n.d. |  |  |
| ZMBN116321 | HB2005.04.15.3 | Hordaland | Bergen | 60.12000 | 5.07000 | 119 | 15/04/2005 | n.d. |  |  |
| ZMBN116322 | HB2005.04.15.3 | Hordaland | Bergen | 60.12000 | 5.07000 | 119 | 15/04/2005 | n.d. | Holotype | Figs 3B, 4B |
| ZMBN116323 | HB2005.04.15.3 | Hordaland | Bergen | 60.12000 | 5.07000 | 119 | 15/04/2005 | n.d. |  |  |
| ZMBN116324 | HB2005.04.15.3 | Hordaland | Bergen | 60.12000 | 5.07000 | 119 | 15/04/2005 | n.d. |  |  |
| ZMBN116325 | HB2005.04.15.3 | Hordaland | Bergen | 60.12000 | 5.07000 | 119 | 15/04/2005 | n.d. |  |  |
| ZMBN116326 | HB2005.04.15.3 | Hordaland | Bergen | 60.12000 | 5.07000 | 119 | 15/04/2005 | n.d. |  |  |
| ZMBN116327 | HM2012/11-12 | Sogn | Aurlandsfjorden | 60.90389 | 7.16813 | 115 | 17/11/2012 | n.d. |  |  |
| ZMBN116328 | HB2007.04.20-trekk112 | Hordaland | Bergen | 60.51018 | 5.19228 | 375 | 20/04/2007 | n.d. |  |  |
| ZMBN116329 | HM2012/07-01 | Sogn-Møre | n.d. | 61.80178 | 5.08135 | 370 - 375 | 20/07/2012 | n.d. |  |  |
| ZMBN116330 | HB2007.06.26-06 | Hordaland | Langenuen | 59.99000 | 5.35000 | 250 | 26/06/2007 | n.d. |  |  |
| ZMBN116331 | HB2007.06.26-06 | Hordaland | Langenuen | 59.99000 | 5.35000 | 250 | 26/06/2007 | n.d. |  |  |
| ZMBN116332 | HB2007.06.26-06 | Hordaland | Langenuen | 59.99000 | 5.35000 | 250 | 26/06/2007 | n.d. |  | Figs 6, 7B |
| **Clade 16** |  |  |  |  |  |  |  |  |  |  |
| **Specimen voucher** | **Site** | **Geographic area** | **Locality** | **Latitude** | **Longitude** | **Depth (m)** | **Collecting date** | **Habitat** | **Remarks** | **Figures** |
| ZMBN116453 | R1158-83 | Finnmark | Finnmark | 71.90850 | 33.44717 | 219 - 220 | 06/08/2013 | n.d. |  |  |
| ZMBN116454 | R1137-77 | Finnmark | Finnmark | 72.57905 | 32.38725 | 271 - 272 | 03/08/2013 | n.d. |  | Fig. 8D, E |
| ZMBN116455 | R1180-86 | Finnmark | Finnmark | 71.61527 | 32.99719 | 305 - 306 | 09/08/2013 | n.d. |  |  |
| ZMBN116456 | R1180-86 | Finnmark | Finnmark | 71.61527 | 32.99719 | 305 - 306 | 09/08/2013 | n.d. |  |  |
| ZMBN116457 | R1180-86 | Finnmark | Finnmark | 71.61527 | 32.99719 | 305 - 306 | 09/08/2013 | n.d. |  |  |
| ZMBN116458 | R1180-86 | Finnmark | Finnmark | 71.61527 | 32.99719 | 305 - 306 | 09/08/2013 | n.d. |  |  |
| ZMBN116459 | R1180-86 | Finnmark | Finnmark | 71.61527 | 32.99719 | 305 - 306 | 09/08/2013 | n.d. |  | Fig. 8A-C, F |
| ZMBN116460 | R1137-456 | Finnmark | Finnmark | 72.58050 | 32.38367 | 271 - 272 | 03/08/2013 | n.d. |  |  |
| ZMBN116461 | R1137-456 | Finnmark | Finnmark | 72.58050 | 32.38367 | 271 - 272 | 03/08/2013 | n.d. |  |  |
| ZMBN116462 | R1137-456 | Finnmark | Finnmark | 72.58050 | 32.38367 | 271 - 272 | 03/08/2013 | n.d. |  |  |
| ZMBN116463 | R1137-456 | Finnmark | Finnmark | 72.58050 | 32.38367 | 271 - 272 | 03/08/2013 | n.d. |  |  |
| ZMBN116464 | R1137-456 | Finnmark | Finnmark | 72.58050 | 32.38367 | 271 - 272 | 03/08/2013 | n.d. |  |  |
| ZMBN116465 | R1137-456 | Finnmark | Finnmark | 72.58050 | 32.38367 | 271 - 272 | 03/08/2013 | n.d. |  |  |
| ZMBN116466 | R1137-456 | Finnmark | Finnmark | 72.58050 | 32.38367 | 271 - 272 | 03/08/2013 | n.d. |  |  |
| ZMBN116467 | R1137-456 | Finnmark | Finnmark | 72.58050 | 32.38367 | 271 - 272 | 03/08/2013 | n.d. |  |  |
| ZMBN116468 | R1137-456 | Finnmark | Finnmark | 72.58050 | 32.38367 | 271 - 272 | 03/08/2013 | n.d. |  |  |
| ZMBN116469 | R1137-456 | Finnmark | Finnmark | 72.58050 | 32.38367 | 271 - 272 | 03/08/2013 | n.d. |  |  |
| ZMBN116470 | R1137-456 | Finnmark | Finnmark | 72.58050 | 32.38367 | 271 - 272 | 03/08/2013 | n.d. |  |  |
| ZMBN116471 | R1137-456 | Finnmark | Finnmark | 72.58050 | 32.38367 | 271 - 272 | 03/08/2013 | n.d. |  |  |
| ZMBN116472 | R942-73 | n.d. | Storegga | 64.39374 | 5.57426 | 814 - 819 | 26/06/2013 | n.d. |  | Fig. 3C |
| ZMBN116473 | R1174-85 | Finnmark | Finnmark | 71.61817 | 32.23133 | 297 - 298 | 08/08/2013 | n.d. |  |  |
| ZMBN116474 | R1180-463 | Finnmark | Finnmark | 71.61416 | 33.00411 | 305 | 09/08/2013 | n.d. |  |  |
| ZMBN116475 | R1180-463 | Finnmark | Finnmark | 71.61416 | 33.00411 | 305 | 09/08/2013 | n.d. |  |  |
| **Clade 24** |  |  |  |  |  |  |  |  |  |  |
| **Specimen voucher** | **Site** | **Geographic area** | **Locality** | **Latitude** | **Longitude** | **Depth (m)** | **Collecting date** | **Habitat** | **Remarks** | **Figures** |
| ZMBN116496 | PS80/355-1 | n.d. | n.d. | 87.92683 | 61.01217 | 4380 | 19/09/2012 | n.d. |  |  |
| ZMBN116497 | PS80/368-1 | n.d. | n.d. | 88.78650 | 56.37200 | 4373 | 23/09/2012 | n.d. |  |  |
| ZMBN116498 | PS80/326-1 | n.d. | n.d. | 81.92700 | 130.91666 | 4038 | 04/09/2012 | n.d. |  | Figs 3D, 4C |
| ZMBN116499 | PS80/368-1 | n.d. | n.d. | 88.78650 | 56.37200 | 4373 | 23/09/2012 | n.d. |  |  |
| ZMBN116500 | PS80/368-1 | n.d. | n.d. | 88.78650 | 56.37200 | 4373 | 23/09/2012 | n.d. |  |  |
| ZMBN116501 | PS80/326-1 | n.d. | n.d. | 81.92700 | 130.91666 | 4038 | 04/09/2012 | n.d. |  | Fig. 13 |
