# Supplementary material for: ﻿A further step towards the characterisation of Terebellides (Annelida, Trichobranchidae) diversity in the Northeast Atlantic, with the description of a new species
Source: Zookeys. 2022 Nov 28;1132:85–126. doi: 10.3897/zookeys.1132.91244 (PMC9836732; doi:10.3897/zookeys.1132.91244)
Supplement: Supplementary material 2 — List of COI sequences considered in present study [file zookeys-1132-085_article-91244__-s002.docx]

Supplementary Material – Table S2

List of COI sequences considered in present study (Group B, C and D), museum vouchers and GenBank accession numbers.

| **Species** | **Type material** | **Specimen voucher** | **Sequence ID** | **GenBank Acc. #** |
| --- | --- | --- | --- | --- |
| *Terebellides shetlandica* | non-type | ZMBN116172 | 825_1 | MG024894 |
| *T. shetlandica* | non-type | ZMBN116173 | 826_1 | MG024895 |
| *T. shetlandica* | non-type | ZMBN116174 | 828_1 | MG024896 |
| *T. shetlandica* | non-type | ZMBN116175 | 830_1 | MG024897 |
| *T. shetlandica* | non-type | ZMBN116176 | 831_1 | MG024898 |
| *T. shetlandica* | non-type | ZMBN116177 | 833_1 | MG024899 |
| *T. shetlandica* | non-type | ZMBN116178 | 834_1 | MG024900 |
| *T. shetlandica* | non-type | ZMBN116179 | 835_1 | MG024901 |
| *T. shetlandica* | non-type | ZMBN116180 | 836_1 | MG024902 |
| *T. shetlandica* | non-type | ZMBN116181 | 837_1 | MG024903 |
| *T. shetlandica* | non-type | GNM15106 | 843_1 | MG024904 |
| *T. shetlandica* | non-type | ZMBN116182 | 856_1 | MG024905 |
| *T. shetlandica* | non-type | ZMBN116183 | 857_1 | MG024906 |
| *T. shetlandica* | non-type | ZMBN116184 | 858_1 | MG024907 |
| *T. shetlandica* | non-type | ZMBN116185 | 1322_1 | MG024908 |
| *T. shetlandica* | non-type | ZMBN116186 | 1323_1 | MG024909 |
| *T. shetlandica* | non-type | ZMBN116187 | 1324_1 | MG024910 |
| *T. shetlandica* | non-type | ZMBN116188 | 1325_1 | MG024911 |
| *T. shetlandica* | non-type | ZMBN116189 | 1326_1 | MG024912 |
| *T. shetlandica* | non-type | ZMBN116190 | 1327_1 | MG024913 |
| *T. shetlandica* | non-type | ZMBN116191 | 1328_1 | MG024914 |
| *T. shetlandica* | non-type | ZMBN116192 | 1329_1 | MG024915 |
| *T. shetlandica* | non-type | ZMBN116193 | 1330_1 | MG024916 |
| *T. shetlandica* | non-type | ZMBN116196 | 1333_1 | MG024917 |
| *T. shetlandica* | non-type | ZMBN116198 | 1335_1 | MG024918 |
| *T. shetlandica* | non-type | ZMBN116199 | 1336_1 | MG024919 |
| *T. shetlandica* | non-type | ZMBN116200 | 1337_1 | MG024920 |
| *T. shetlandica* | non-type | ZMBN116201 | 1338_1 | MG024921 |
| *T. shetlandica* | non-type | ZMBN116202 | 1339_1 | MG024922 |
| *T. shetlandica* | non-type | ZMBN116203 | 1340_1 | MG024923 |
| *T. shetlandica* | non-type | ZMBN116205 | 1342_1 | MG024924 |
| *T. shetlandica* | non-type | ZMBN116206 | 1344_1 | MG024925 |
| *T. shetlandica* | non-type | ZMBN116207 | 1938_1 | MG024926 |
| *T. shetlandica* | non-type | ZMBN116208 | 1939_1 | MG024927 |
| *T. shetlandica* | non-type | ZMBN116209 | 1940_1 | MG024928 |
| *T. shetlandica* | non-type | ZMBN116211 | 1945_1 | MG024929 |
| *T. shetlandica* | non-type | ZMBN116212 | 1947_1 | MG024930 |
| *T. shetlandica* | non-type | ZMBN116213 | 1948_1 | MG024931 |
| *T. shetlandica* | non-type | ZMBN116214 | 1949_1 | MG024932 |
| *T. shetlandica* | non-type | ZMBN116215 | 1950_1 | MG024933 |
| *T. shetlandica* | non-type | ZMBN116216 | 1951_1 | MG024934 |
| *T. shetlandica* | non-type | ZMBN116217 | 1952_1 | MG024935 |
| *T. shetlandica* | non-type | GNM14649 | 2241_1 | MG024936 |
| *T. shetlandica* | non-type | ZMBN116221 | 2439_1 | MG024937 |
| *T. shetlandica* | non-type | ZMBN116222 | 2440_1 | MG024938 |
| *T. shetlandica* | non-type | ZMBN116223 | 2441_1 | MG024939 |
| *T. shetlandica* | non-type | ZMBN116224 | 2444_1 | MG024940 |
| *T. shetlandica* | non-type | ZMBN116225 | 2445_1 | MG024941 |
| *T. shetlandica* | non-type | ZMBN116226 | 2446_1 | MG024942 |
| *T. shetlandica* | non-type | ZMBN116228 | 2453_1 | MG024943 |
| *T. shetlandica* | non-type | ZMBN116232 | 2794_1 | MG024944 |
| *T. shetlandica* | non-type | ZMBN116233 | 2796_1 | MG024945 |
| *T. shetlandica* | non-type | ZMBN116235 | 2839_1 | MG024946 |
| *T. shetlandica* | non-type | ZMBN116237 | 2841_1 | MG024947 |
| *T. shetlandica* | non-type | ZMBN116238 | 2860_1 | MG024948 |
| *T. shetlandica* | non-type | ZMBN116239 | 2861_1 | MG024949 |
| *T. shetlandica* | non-type | ZMBN116240 | 2862_1 | MG024950 |
| *T. shetlandica* | non-type | ZMBN116241 | 2871_1 | MG024951 |
| *T. shetlandica* | non-type | ZMBN116242 | 2909_1 | MG024952 |
| *T. shetlandica* | non-type | ZMBN116243 | 2911_1 | MG024953 |
| *T. shetlandica* | non-type | NTNU-VM61384 | T05_1 | MG024954 |
| *T. shetlandica* | non-type | NTNU-VM61385 | T06_1 | MG024955 |
| *T. shetlandica* | non-type | NTNU-VM59990 | TB29_1 | MG024956 |
| *Terebellides williamsae* | non-type | GNM15107 | 841_2 | MG024957 |
| *T. williamsae* | non-type | GNM15108 | 844_2 | MG024958 |
| *T. williamsae* | non-type | GNM15109 | 1311_2 | MG024959 |
| *T. williamsae* | non-type | ZMBN116272 | 1987_2 | MG024960 |
| *T. williamsae* | non-type | ZMBN116244 | 2180_2 | MG024961 |
| *T. williamsae* | non-type | ZMBN116245 | 2181_2 | MG024962 |
| *T. williamsae* | non-type | ZMBN116246 | 2182_2 | MG024963 |
| *T. williamsae* | non-type | ZMBN116247 | 2185_2 | MG024964 |
| *T. williamsae* | non-type | ZMBN116248 | 2187_2 | MG024965 |
| *T. williamsae* | non-type | GNM14639 | 2216_2 | MG024966 |
| *T. williamsae* | non-type | SMF24679 | 2271_2 | MG024967 |
| *T. williamsae* | non-type | SMF24673 | 2272_2 | MG024968 |
| *T. williamsae* | non-type | ZMBN116249 | 2322_2 | MG024969 |
| *T. williamsae* | non-type | ZMBN116250 | 2326_2 | MG024970 |
| *T. williamsae* | non-type | ZMBN116251 | 2328_2 | MG024971 |
| *T. williamsae* | non-type | ZMBN116252 | 2331_2 | MG024972 |
| *T. williamsae* | non-type | ZMBN116253 | 2332_2 | MG024973 |
| *T. williamsae* | non-type | ZMBN116254 | 2333_2 | MG024974 |
| *T. williamsae* | non-type | ZMBN116256 | 2338_2 | MG024975 |
| *T. williamsae* | non-type | ZMBN116257 | 2351_2 | MG024976 |
| *T. williamsae* | non-type | ZMBN116258 | 2352_2 | MG024977 |
| *T. williamsae* | non-type | ZMBN116259 | 2353_2 | MG024978 |
| *T. williamsae* | non-type | ZMBN116260 | 2354_2 | MG024979 |
| *T. williamsae* | non-type | ZMBN116261 | 2360_2 | MG024980 |
| *T. williamsae* | non-type | ZMBN116262 | 2367_2 | MG024981 |
| *T. williamsae* | non-type | ZMBN116263 | 2368_2 | MG024982 |
| *T. williamsae* | non-type | ZMBN116264 | 2369_2 | MG024983 |
| *T. williamsae* | non-type | ZMBN116265 | 2370_2 | MG024984 |
| *T. williamsae* | non-type | ZMBN116267 | 2378_2 | MG024985 |
| *T. williamsae* | non-type | ZMBN116268 | 2381_2 | MG024986 |
| *T. williamsae* | non-type | ZMBN116269 | 2382_2 | MG024987 |
| *T. williamsae* | non-type | NTNU-VM68253 | POLYNOR88_2 | MG024988 |
| *Terebellides gracilis* | non-type | GNM15110 | 1207_3 | MG024989 |
| *T. gracilis* | non-type | GNM15111 | 1310_3 | MG024990 |
| *T. gracilis* | non-type | SMF24669 | 2275_3 | MG024991 |
| *T. gracilis* | non-type | SMF24683 | 2285_3 | MG024992 |
| *T. gracilis* | non-type | SMF24667 | 2287_3 | MG024993 |
| *T. gracilis* | non-type | ZMBN116273 | 2380_3 | MG024994 |
| *T. gracilis* | non-type | SMF24692 | 2463_3 | MG024995 |
| *T. gracilis* | non-type | SMF24665 | 2464_3 | MG024996 |
| *T. gracilis* | non-type | SMF24668 | 2465_3 | MG024997 |
| *T. gracilis* | non-type | NTNU-VM68196 | 2480_3 | MG024998 |
| *T. gracilis* | non-type | ZMBN116276 | 2812_3 | MG024999 |
| *T. gracilis* | non-type | ZMBN116277 | 2814_3 | MG025000 |
| *T. gracilis* | non-type | ZMBN116278 | 2872_3 | MG025001 |
| *T. gracilis* | non-type | ZMBN116279 | 2873_3 | MG025002 |
| *T. gracilis* | non-type | ZMBN116280 | 2874_3 | MG025003 |
| *T. gracilis* | non-type | ZMBN116281 | 2876_3 | MG025004 |
| *T. gracilis* | non-type | ZMBN116282 | 2877_3 | MG025005 |
| *T. gracilis* | non-type | ZMBN116283 | 2878_3 | MG025006 |
| *T. gracilis* | non-type | ZMBN116284 | 2879_3 | MG025007 |
| *T. gracilis* | non-type | ZMBN116285 | 2880_3 | MG025008 |
| *T. gracilis* | non-type | ZMBN116286 | 2881_3 | MG025009 |
| *T. gracilis* | non-type | ZMBN116287 | 2882_3 | MG025010 |
| *T. gracilis* | non-type | ZMBN116288 | 2883_3 | MG025011 |
| *T. gracilis* | non-type | ZMBN116289 | 2884_3 | MG025012 |
| *T. gracilis* | non-type | ZMBN116290 | 2885_3 | MG025013 |
| *T. gracilis* | non-type | ZMBN116291 | 2886_3 | MG025014 |
| *T. gracilis* | non-type | ZMBN116292 | 2887_3 | MG025015 |
| *T. gracilis* | non-type | ZMBN116293 | 2888_3 | MG025016 |
| *T. gracilis* | non-type | ZMBN116294 | 2889_3 | MG025017 |
| *T. gracilis* | non-type | ZMBN116295 | 2890_3 | MG025018 |
| *T. gracilis* | non-type | ZMBN116296 | 2891_3 | MG025019 |
| *T. gracilis* | non-type | ZMBN116297 | 2901_3 | MG025020 |
| *T. gracilis* | non-type | ZMBN116299 | 2906_3 | MG025021 |
| *T. gracilis* | non-type | ZMBN116300 | 2907_3 | MG025022 |
| *T. gracilis* | non-type | ZMBN116301 | 2908_3 | MG025023 |
| *T. gracilis* | non-type | ZMBN116302 | 2910_3 | MG025024 |
| *T. gracilis* | non-type | ZMBN116303 | 2912_3 | MG025025 |
| *T. gracilis* | non-type | ZMBN116304 | 2913_3 | MG025026 |
| *T. gracilis* | non-type | ZMBN116305 | 2915_3 | MG025027 |
| *T. gracilis* | non-type | ZMBN116306 | 2916_3 | MG025028 |
| *T. gracilis* | non-type | ZMBN116307 | 2917_3 | MG025029 |
| *T. gracilis* | non-type | ZMBN116308 | 2922_3 | MG025030 |
| *T. gracilis* | non-type | ZMBN116309 | 2923_3 | MG025031 |
| *T. gracilis* | non-type | ZMBN116311 | 2929_3 | MG025032 |
| *T. gracilis* | non-type | ZMBN116312 | 2930_3 | MG025033 |
| *T. gracilis* | non-type | ZMBN116313 | 2931_3 | MG025034 |
| *T. gracilis* | non-type | ZMBN116314 | 2932_3 | MG025035 |
| *T. gracilis* | non-type | ZMBN116315 | 2933_3 | MG025036 |
| *T. gracilis* | non-type | ZMBN116316 | 2934_3 | MG025037 |
| *T. lavesquei* sp. nov. | non-type | ZMBN116317 | 2935_3 | MG025038 |
| *T. lavesquei* sp. nov. | Paratype | GNM15112 | 840_5 | MG025053 |
| *T. lavesquei* sp. nov. | non-type - | GNM15113 | 842_5 | MG025054 |
| *T. lavesquei* sp. nov. | Paratype | ZMBN116319 | 2777_5 | MG025055 |
| *T. lavesquei* sp. nov. | Paratype | ZMBN116320 | 2778_5 | MG025056 |
| *T. lavesquei* sp. nov. | Paratype | ZMBN116321 | 2779_5 | MG025057 |
| *T. lavesquei* sp. nov. | Holotype | ZMBN116322 | 2780_5 | MG025058 |
| *T. lavesquei* sp. nov. | Paratype | ZMBN116323 | 2790_5 | MG025059 |
| *T. lavesquei* sp. nov. | Paratype | ZMBN116324 | 2791_5 | MG025060 |
| *T. lavesquei* sp. nov. | Paratype | ZMBN116325 | 2792_5 | MG025061 |
| *T. lavesquei* sp. nov. | Paratype | ZMBN116326 | 2793_5 | MG025062 |
| *T. lavesquei* sp. nov. | Paratype | ZMBN116327 | 2900_5 | MG025063 |
| *T. lavesquei* sp. nov. | Paratype | ZMBN116328 | 2904_5 | MG025064 |
| *T. lavesquei* sp. nov. | Paratype | ZMBN116329 | 2918_5 | MG025065 |
| *T. lavesquei* sp. nov. | Paratype | ZMBN116330 | 2926_5 | MG025066 |
| *T. lavesquei* sp. nov. | Paratype | ZMBN116331 | 2927_5 | MG025067 |
| *T. lavesquei* sp. nov. | Paratype | ZMBN116332 | 2928_5 | MG025068 |
| *T. lavesquei* sp. nov. | Paratype | NTNU-VM68252 | POLYNOR087_5 | MG025069 |
| *T. lavesquei* sp. nov. | Paratype | NTNU-VM61386 | T02_5 | MG025070 |
| *T. lavesquei* sp. nov. | Paratype | NTNU-VM61387 | T04_5 | MG025071 |
| *T. atlantis* | non-type | SMF24677 | 2267_16 | MG025258 |
| *T. atlantis* | non-type | SMF24678 | 2268_16 | MG025259 |
| *T. atlantis* | non-type | SMF24663 | 2269_16 | MG025260 |
| *T. atlantis* | non-type | SMF24671 | 2270_16 | MG025261 |
| *T. atlantis* | non-type | SMF24646 | 2279_16 | MG025262 |
| *T. atlantis* | non-type | SMF24681 | 2280_16 | MG025263 |
| *T. atlantis* | non-type | SMF24682 | 2284_16 | MG025264 |
| *T. atlantis* | non-type | SMF24674 | 2288_16 | MG025265 |
| *T. atlantis* | non-type | SMF24643 | 2289_16 | MG025266 |
| *T. atlantis* | non-type | SMF24645 | 2290_16 | MG025267 |
| *T. atlantis* | non-type | SMF24652 | 2294_16 | MG025268 |
| *T. atlantis* | non-type | SMF24664 | 2295_16 | MG025269 |
| *T. atlantis* | non-type | SMF24686 | 2296_16 | MG025270 |
| *T. atlantis* | non-type | SMF24655 | 2297_16 | MG025271 |
| *T. atlantis* | non-type | SMF24649 | 2298_16 | MG025272 |
| *T. atlantis* | non-type | SMF24687 | 2299_16 | MG025273 |
| *T. atlantis* | non-type | SMF24642 | 2300_16 | MG025274 |
| *T. atlantis* | non-type | SMF24662 | 2303_16 | MG025275 |
| *T. atlantis* | non-type | SMF24660 | 2305_16 | MG025276 |
| *T. atlantis* | non-type | SMF24653 | 2306_16 | MG025277 |
| *T. atlantis* | non-type | SMF24641 | 2307_16 | MG025278 |
| *T. atlantis* | non-type | SMF24651 | 2308_16 | MG025279 |
| *T. atlantis* | non-type | SMF24656 | 2309_16 | MG025280 |
| *T. atlantis* | non-type | SMF24639 | 2310_16 | MG025281 |
| *T. atlantis* | non-type | SMF24658 | 2311_16 | MG025282 |
| *T. atlantis* | non-type | SMF24691 | 2315_16 | MG025283 |
| *T. atlantis* | non-type | SMF24661 | 2316_16 | MG025284 |
| *T. atlantis* | non-type | ZMBN116453 | 2325_16 | MG025285 |
| *T. atlantis* | non-type | ZMBN116454 | 2330_16 | MG025286 |
| *T. atlantis* | non-type | ZMBN116455 | 2339_16 | MG025287 |
| *T. atlantis* | non-type | ZMBN116456 | 2340_16 | MG025288 |
| *T. atlantis* | non-type | ZMBN116458 | 2344_16 | MG025289 |
| *T. atlantis* | non-type | ZMBN116459 | 2345_16 | MG025290 |
| *T. atlantis* | non-type | ZMBN116460 | 2355_16 | MG025291 |
| *T. atlantis* | non-type | ZMBN116461 | 2356_16 | MG025292 |
| *T. atlantis* | non-type | ZMBN116462 | 2357_16 | MG025293 |
| *T. atlantis* | non-type | ZMBN116463 | 2358_16 | MG025294 |
| *T. atlantis* | non-type | ZMBN116464 | 2359_16 | MG025295 |
| *T. atlantis* | non-type | ZMBN116465 | 2361_16 | MG025296 |
| *T. atlantis* | non-type | ZMBN116466 | 2362_16 | MG025297 |
| *T. atlantis* | non-type | ZMBN116467 | 2363_16 | MG025298 |
| *T. atlantis* | non-type | ZMBN116468 | 2364_16 | MG025299 |
| *T. atlantis* | non-type | ZMBN116469 | 2365_16 | MG025300 |
| *T. atlantis* | non-type | ZMBN116470 | 2366_16 | MG025301 |
| *T. atlantis* | non-type | ZMBN116471 | 2373_16 | MG025302 |
| *T. atlantis* | non-type | ZMBN116472 | 2374_16 | MG025303 |
| *T. atlantis* | non-type | ZMBN116474 | 2389_16 | MG025304 |
| *T. atlantis* | non-type | SMF24693 | 2466_16 | MG025305 |
| *T. atlantis* | non-type | SMF24644 | 2467_16 | MG025306 |
| *T. atlantis* | non-type |  | 2468_16 | MG025307 |
| *T. atlantis* | non-type | SMF24650 | 2469_16 | MG025308 |
| *T. atlantis* | non-type | SMF24654 | 2470_16 | MG025309 |
| *T. atlantis* | non-type | SMF24640 | 2471_16 | MG025310 |
| *T. atlantis* | non-type | SMF24648 | 2472_16 | MG025311 |
| *T. atlantis* | non-type | SMF24657 | 2474_16 | MG025312 |
| *Terebellides irinae* | non-type | ZMBN116496 | 2864_24 | MG025340 |
| *T. irinae* | non-type | ZMBN116497 | 2865_24 | MG025341 |
| *T. irinae* | non-type | ZMBN116500 | 2866_24 | MG025342 |
| *T. irinae* | non-type | ZMBN116498 | 2867_24 | MG025343 |
| *T. irinae* | non-type | ZMBN116499 | 2869_24 | MG025344 |
